# An Outer Membrane Vesicle‐Based Permeation Assay (OMPA) for Assessing Bacterial Bioavailability

**DOI:** 10.1002/adhm.202101180

**Published:** 2021-10-24

**Authors:** Robert Richter, Mohamed A. M. Kamal, Marcus Koch, Bart‐Jan Niebuur, Anna‐Lena Huber, Adriely Goes, Carsten Volz, Julia Vergalli, Tobias Kraus, Rolf Müller, Nicole Schneider‐Daum, Gregor Fuhrmann, Jean‐Marie Pagès, Claus‐Michael Lehr

**Affiliations:** ^1^ Helmholtz Centre for Infection Research Helmholtz Institute for Pharmaceutical Research Saarland Campus E8.1 Saarbrücken 66123 Germany; ^2^ Saarland University Department of Pharmacy Campus E8.1 Saarbrücken 66123 Germany; ^3^ INM – Leibniz Institute for New Materials Campus D2.2 Saarbrücken 66123 Germany; ^4^ UMR_MD1 U‐1261 Aix‐Marseille Université INSERM IRBA MCT Faculté de Pharmacie 27 Boulevard Jean Moulin Marseille 13005 France; ^5^ Colloid and Interface Chemistry Saarland University Campus D2.2 Saarbrücken 66123 Germany

**Keywords:** antimicrobial resistance, bacterial bioavailability, drug optimization, extracellular vesicles, in vitro studies, membrane permeation models

## Abstract

When searching for new antibiotics against Gram‐negative bacterial infections, a better understanding of the permeability across the cell envelope and tools to discriminate high from low bacterial bioavailability compounds are urgently needed. Inspired by the phospholipid vesicle‐based permeation assay (PVPA), which is designed to predict non‐facilitated permeation across phospholipid membranes, outer membrane vesicles (OMVs) of *Escherichia coli* either enriched or deficient of porins are employed to coat filter supports for predicting drug uptake across the complex cell envelope. OMVs and the obtained in vitro model are structurally and functionally characterized using cryo‐TEM, SEM, CLSM, SAXS, and light scattering techniques. In vitro permeability, obtained from the membrane model for a set of nine antibiotics, correlates with reported in bacterio accumulation data and allows to discriminate high from low accumulating antibiotics. In contrast, the correlation of the same data set generated by liposome‐based comparator membranes is poor. This better correlation of the OMV‐derived membranes points to the importance of hydrophilic membrane components, such as lipopolysaccharides and porins, since those features are lacking in liposomal comparator membranes. This approach can offer in the future a high throughput screening tool with high predictive capacity or can help to identify compound‐ and bacteria‐specific passive uptake pathways.

## Graphical abstract

A filter support‐based model of the Gram‐negative bacterial outer membrane is developed employing outer membrane vesicles from a porin‐containing and porin‐deficient *Escherichia coli* strain. Permeation data obtained from outer membrane vesicle‐derived models are in better agreement with reported data on bacterial bioavailability than permeation data obtained from liposomal comparator models.

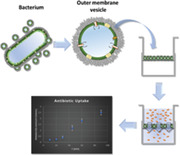

## 1 Introduction

New antibiotic classes against Gram‐negative bacterial infections are urgently needed to cope with the steadily emerging antimicrobial resistance. Within the last 30 years, no new class has been deployed to the market^[^ ^1^ ^]^ and the pipeline of antibiotics with anti‐Gram‐negative spectrum has been drying out,^[^ ^2^ ^]^ which caused the WHO to include mainly Gram‐negative bacteria into their pathogen priority list.^[^ ^3^ ^]^ In order to keep the pipeline filled with promising candidates, it is important to employ powerful and discriminating in vitro assays already at an early stage of drug development. The Gram‐negative bacterial cell envelope is a part of the cell that has constantly been challenging drug developers, leading to compounds with good activity at the target, but no activity inside the living bacterium.^[^ ^4^ ^]^ The complex structure of the envelope has been subjected to extensive research and reports. In contrast to Gram‐positive bacteria, the envelope of Gram‐negative bacteria is composed of two membranes, of which the outer membrane (OM) is asymmetric containing mainly phospholipids (PLs) on its inner and lipopolysaccharides (LPS) on its outer leaflet as well as outer membrane proteins with partially channel‐like structure (porins). The ensemble of LPS and porins forms an effective barrier to most lipophilic as well as hydrophilic compounds.^[^ ^5^, ^6^, ^7^ ^]^ To help drug developers also optimize their structures toward better cell penetration and enhanced bacterial bioavailability,^[^ ^8^ ^]^ various approaches have been undertaken, ranging from cell‐based assays,^[^ ^9^, ^10^, ^11^, ^12^, ^13^ ^]^ vesicle swelling assays,^[^ ^14^, ^15^ ^]^ electrophysiological assays^[^ ^16^, ^17^ ^]^ to PAMPA‐like filter support‐based assays coated with different biomaterials.^[^ ^18^, ^19^, ^20^ ^]^ Only the latter assay type currently offers the opportunity to be employed in high throughput screening. However, the so far employed materials either mimic non‐facilitated uptake^[^ ^18^, ^19^ ^]^ or mainly facilitated uptake^[^ ^20^ ^]^ while employing either artificial PLs or physiologically irrelevant hydrogels. By introducing natural bacterial membrane components such as LPS and porins into such an assay via OMVs, predictive capacity and strain specificity could be increased while the impact of several components on antibiotic translocation can be observed. This would make such assays even more useful in early anti‐Gram‐negative drug development, especially in the phase of lead identification and lead optimization.^[^ ^21^ ^]^

Extracellular vesicles (EV) are a biomaterial constitutively shed by virtually all cells to their environment.^[^ ^22^ ^]^ Although their concept can be even traced back as far as to Charles Darwin,^[^ ^23^ ^]^ these spherical membrane structures have only recently become a popular focus of research related to diagnosis,^[^ ^24^, ^25^ ^]^ cellular behavior and interaction,^[^ ^26^, ^27^ ^]^ pathogen virulence as well as drug delivery.^[^ ^21^, ^28^, ^29^ ^]^ Depending on their origin and composition, EVs fulfill different purposes, such as cell‐to‐cell communication, transfer of resistance genes as well as virulence factors and defense,^[^ ^30^ ^]^

Outer membrane vesicles (OMVs) are one type of EVs, which originate from the OM of Gram‐negative bacteria and provide them with unique characteristics. One important feature is the presence of OM proteins, of which porins – protein channels of the OM, allowing for a selective translocation into the Gram‐negative bacterial cell – proved to significantly rule overall bacterial bioavailability and eventually antibiotic activity of small polar molecules.^[^ ^31^, ^32^, ^33^ ^]^ Furthermore, OMVs also contain LPS and contain cargo, such as enzymes, nucleic acids, or peptidoglycan.^[^ ^21^ ^]^ Since the OM of Gram‐negative bacteria due to the presence of porins and LPS appears to be the major delimiter of antibiotic permeation,^[^ ^32^, ^34^ ^]^ OMVs seem eligible also for repurposing as an in vitro model for drug permeation studies. Nakae et al.,^[^ ^35^ ^]^ Ferreira et al.^[^ ^15^ ^]^ as well as Wang et al.^[^ ^17^ ^]^ employed already bacterial membrane vesicles to investigate the porin‐mediated uptake of saccharides or antibiotics, respectively. OMVs can be considered to some degree as naturally derived proteoliposomes, but with higher complexity regarding their structure and content.^[^ ^21^, ^36^ ^]^

**Figure 1:**
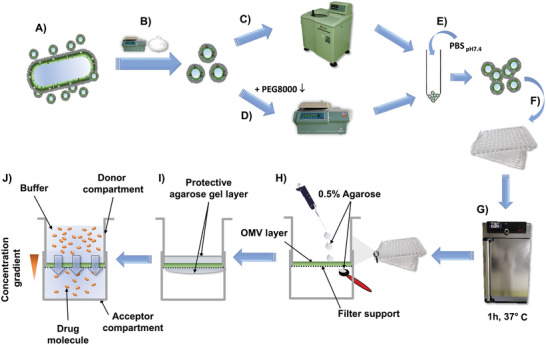
Workflow for the preparation of an OMV‐based membrane model. After culturing bacteria in lysogeny broth A), they are separated from the supernatant by centrifugation and sterile filtration B). The supernatant is either subjected to ultracentrifugation C) or polyethylene glycol precipitation followed by normal centrifugation D). The pellet is resuspended in phosphate‐buffered saline E) and the suspension is given on top of the wells with polycarbonate filter supports F) followed by drying of the suspension at 37°C G), which can be repeated multiple times. The eventually obtained coating H) is covered in a 0.5% w/v agarose hydrogel to protect it from mechanical stress and to moisturize it I), enabling permeation assays J).

Hence, we hypothesize that employing them in a fashion of a phospholipid vesicle‐based permeation assay (PVPA)^[^ ^37^ ^]^ yields considerable advantages in predicting antibiotic uptake and bacterial bioavailability. PVPAs based on liposomes made of egg phosphatidylcholine (PC) have shown to successfully predict intestinal absorption of various oral drugs.^[^ ^38^ ^]^ Moreover, such assays are attractive since they are easy to prepare as well as potentially scalable toward high throughput methods. Employing OMVs with their unique and cell‐specific features instead of liposomes^[^ ^18^, ^19^ ^]^ may enable such an assay to allow for better predictions regarding the uptake of antibiotics into Gram‐negative bacteria and bacterial bioavailability while keeping the opportunity of higher compound throughput. As protocols for liposomal coating of filter supports exist,^[^ ^37^ ^]^ these can be easily adapted to OMVs to achieve optimal functionality of such a so‐called OMV‐based permeation assay (OMPA). (**Figure** **1**) In this study, we cultured different Gram‐negative bacterial species, compared different OMV isolation protocols, and characterized the obtained OMVs as well as previously employed comparator liposomes^[^ ^18^, ^37^, ^39^ ^]^ regarding their yield as well as physicochemical properties using nanoparticle tracking analysis (NTA), zeta‐sizing, and cryo‐TEM. We investigated the presence of selected OM proteins by western blot and MALDI‐TOF‐MS while the membrane morphology was assessed by SEM as well as small‐ and wide‐angle x‐ray spectroscopy (SAXS and WAXS, resp.). Confocal laser‐scanning microscopy (CLSM) and fluorescence recovery after photobleaching (FRAP) assays served to investigate biophysical properties of the membrane, such as swelling and membrane fluidity. The obtained OMV‐based membrane model at its final state and liposome‐based comparator models were subjected to permeation studies using commercially available antibiotics. The obtained permeability data were compared to reported data on whole‐cell accumulation. Eventually, they were also compared among different vesicle types, to assess the impact of different OMV components, such as porins, lipopolysaccharides, and PL composition.

## 2 Results

### 2.1 Optimization of OMV Isolation

Maximizing the yield of OMVs is essential for an adequate coating of the filter support. This can be achieved in various ways. In order to explore the impact of different variations on the vesicle yield within a suitable analytical working range, we chose the reportedly hypervesiculating strain *Escherichia coli* BL21 DE3.^[^ ^40^ ^]^ First, the impact of the duration of the liquid culture was investigated. We hypothesized that an extended culturing time of seven versus two days would cause starvation to the bacteria and result in a further increase in the OMV production.^[^ ^41^ ^]^ Indeed, a seven‐day culture could increase the vesicle yield significantly. (Figure S1A, Supporting Information) We then decided to routinely culture strain BL21 DE3 for one week to obtain the maximum yield.

**Figure 2:**
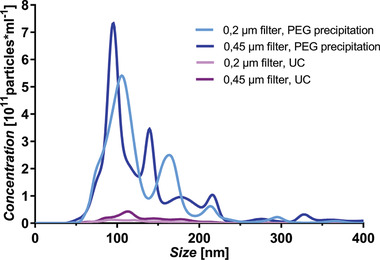
Vesicle yield and size distribution following different isolation protocols. Precipitation with 33% w/w PEG8000 solution (blue lines) resulted in a higher yield of OMV than ultracentrifugation (UC, pink lines), while the pore size of the filter altered the size distribution but not the overall yield. Each line represents mean size distribution as obtained from nanoparticle tracking analysis. *N* = 9 from three independent experiments.

Another crucial factor is the isolation method, of which various are reported.^[^ ^42^, ^43^ ^]^ In general, there is the tendency that methods leading to higher EV purity result in a lower yield and vice versa. Here, we selected and compared two popular methods, namely ultracentrifugation (UC) and vesicle precipitation by a highly concentrated (33% w/w) PEG 8000 solution. As previously described, the precipitation technique clearly resulted in a higher yield of OMV than UC.^[^ ^44^ ^]^ Filter pore sizes of 0.2 and 0.45 µm, however, did not lead to significant differences (**Figure** **2**) in the yield but slightly shifted the particle size distribution. Notably, maxima beyond 200 nm are visible at similar locations for 0.45 and 0.2 µm pore sizes, respectively, which may indicate the formation of OMV agglomerates.

Considering the slightly higher yield when employing a 0.45 µm pore‐sized filter and the lower pressure required for the filtration process, we chose to combine the filtration through filters with 0.45 µm pore diameter and the OMV precipitation by a PEG 8000 solution.

**Figure 3:**
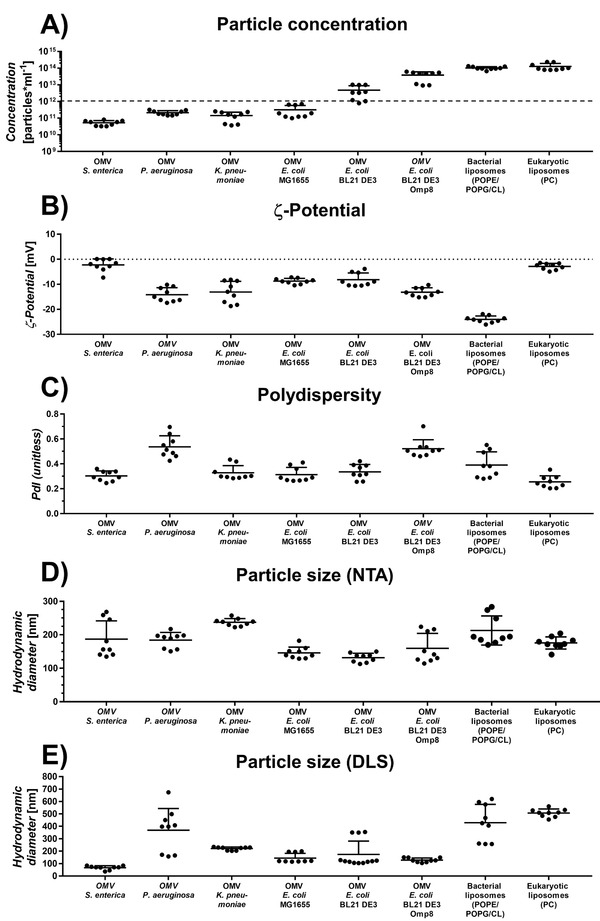
Physicochemical properties of selected outer membrane vesicles (OMVs) and comparator liposomes in phosphate‐buffered saline (pH 7.4). There are species‐ and strain‐dependent dramatic differences between the amount of shed OMV A). The dashed line at 10^12^ particles mL^−1^ indicates the minimum yield required for subsequent coating. All vesicles show a slightly negative species‐ and strain‐specific zeta‐potential B), while the polydispersity is throughout all investigated vesicles high C). Hydrodynamic diameters were determined by D) nanoparticle tracking analysis (NTA) and E) dynamic light scattering (DLS). Horizontal lines represent mean + SD. Data obtained from three independent experiments with three technical replicates each. A one‐way ANOVA was performed with Tukey's multiple comparisons post‐hoc analysis (Section S2, Supporting Information).

Apart from *E. coli* BL21 DE3, we included further bacterial species and strains for comparison, isolated their OMVs, and characterized them physicochemically. In addition, we produced comparator liposomes, composed of either PLs, typical of Gram‐negative bacteria (POPE, POPG, Cardiolipin 7:2:1 (m/m/m)) or egg PC, typical of mammalian cell membranes as described earlier^[^ ^18^ ^]^ (**Figure** **3**).

Notably, *E. coli* BL21 DE3 produces ten times more OMVs than the wild‐type strain *E. coli* MG1655. (Figure 3A) Other bacterial species, such as *Salmonella enterica*, *Pseudomonas aeruginosa* and *Klebsiella pneumoniae* produce vesicles at a level that is similar to *E. coli* MG1655 or even lower. In contrast to that, *E. coli* BL21 DE3 Omp8 being devoid of OM proteins OmpF, OmpC, OmpA, and LamB^[^ ^45^ ^]^ produces vesicles at considerable yield, which is similar to the concentrations obtained from our liposome production protocol. In contrast to *E. coli* BL21 DE3, no significant difference was obtained between a two‐day and seven‐day culture of *E. coli* BL21 DE3 Omp8 (Figure S1B, Supporting Information). Therefore, we decided to limit the culture time of *E. coli* BL21 DE3 Omp8 to two days in order to avoid the loss of the transposon tn5, which suppresses OmpF expression.^[^ ^45^ ^]^

### 2.2 Physicochemical Vesicle Characterization

**Figure 4:**
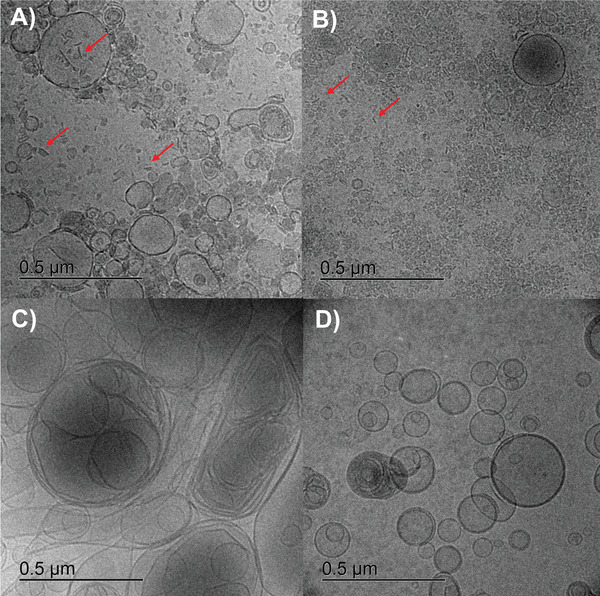
Cryo‐TEM micrographs of *E. coli* BL21 DE3 OMV A) and selected comparator vesicles of *E. coli* BL21 DE3 Omp8 B), bacterial liposomes C) and eukaryotic liposomes D) at undiluted state. Non‐vesicular lamellar structures are indicated by red arrows.

The *ζ*‐potential of OMVs varies between the investigated bacterial species as well as strains and with respect to *P. aeruginosa* and *K. pneumoniae* even between vesicle batches. Strikingly, OMVs of the intracellular pathogen *S. enterica* have a *ζ*‐potential near 0 mV, which is comparable to the eukaryotic liposomes, and is probably due to a high content of the zwitterionic PL phosphatidylethanolamine, as is characteristic for the parental OM^[^ ^46^ ^]^ and may aid the infection of eukaryotic cells. OMVs of *P. aeruginosa* and *K. pneumoniae* have a *ζ*‐potential, which is significantly lower than of *E. coli* MG1655, BL21 DE3 and *S. enterica* (Section S2, Supporting Information). It is worth mentioning that this ranking somewhat matches previously reported relative amounts of negatively charged PLs found in the respective bacteria.^[^ ^46^, ^47^, ^48^ ^]^ The POPG and cardiolipin‐rich bacterial liposomes showed the lowest *ζ*‐potential, while – as expected – the *ζ*‐potential of the eukaryotic liposomes was near zero. These studies indicate that the PL composition of vesicles determines their surface charge in a considerable manner. Since OMVs are natural products with many different purposes, it is expectable that their size varies resulting in a relatively high polydispersity index. (Figure 3C) By comparing the vesicle sizes obtained either by nanoparticle tracking analysis (NTA) or dynamic light scattering (DLS) (Figure 3D,E), we noticed discrepancies regarding OMVs from *S. enterica*, *P. aeruginosa* as well as the liposomal formulations. These discrepancies expand to *E. coli* OMVs when comparing NTA and DLS data to cryo‐TEM micrographs (**Figure** **4**).

The latter show a dramatic difference between OMV sizes of *E. coli* BL21 DE3 and BL21 DE3 Omp8, which could not be revealed by NTA and DLS. Obviously, the extremely small OMVs of *E. coli* BL21 DE3 Omp8 tend to form agglomerates, making it difficult to measure the correct size by light scattering techniques. Cryo‐TEM also revealed the different vesicle morphologies. Notably, OMVs of our *E. coli* strains are accompanied by partially stacked lamellar membrane‐like structures independently from their isolation method (Figure 4A,B and Figure S2A,B: Supporting Information), suggesting them to be remnants of collapsed OMVs or flagella.^[^ ^49^ ^]^ Those observations were also made earlier on other Gram‐negative as well as ‐positive species.^[^ ^50^, ^51^ ^]^

Furthermore, earlier reported outer‐inner membrane vesicles could be observed in low amounts in vesicle suspensions of *E. coli* BL21 DE3 and BL21 DE3 Omp8 (Figure S2A,B: Supporting Information).^[^ ^52^ ^]^

However, multilamellar structures were much more frequently observed among the liposomal formulations. Notably, bacterial comparator liposomes composed of POPE, POPG and cardiolipin appear less spherical than eukaryotic liposomes made of egg PC (Figure 4C,D). This phenomenon is independent of their concentration (Section S2D,E: Supporting Information).

When observing vesicles of *E. coli* BL21 DE3 by SEM, we found that at regions of higher concentrated OMV, fusion occurred during the drying process (Figure S3, Supporting Information), which could be favorable for the envisaged coating process.

OMVs of *E. coli* BL21 DE3 and BL21 DE3 Omp8 were further investigated regarding their expression of OM proteins employing either MALDI‐TOF‐MS or western blot (Figure S4, Supporting Information). The presence of OmpA, OmpC, and OmpF on OMVs of *E. coli* BL21 DE3 Omp8 would indicate an unwanted expression of these outer membrane proteins either due to mutation or tn5 transposon loss, which would render this strain inappropriate for a porin‐less control vesicle. In contrast, the presence of at least OmpF is required for OMVs of *E. coli* BL21 DE3 to obtain a porin‐containing permeation model. Lysed *E. coli* MG1655 and its OMVs were employed as comparator material to evaluate the abundance of the respective porins in the wild‐type organism. To elucidate the protein composition, SDS‐PAGE was employed to separate OM proteins of interest (OmpA, OmpC, OmpF) from bacterial lysate of *E. coli* MG1655 as well as from OMVs of *E. coli* MG1655, BL21 DE3, and BL21 DE3 Omp8. Their bands were cut off the gel and extracted for MALDI‐TOF‐MS analysis, where only for *E. coli* BL21 DE3, two proteins, namely OmpF (Mascot score: 74) and OmpA (Mascot score: 36) could be detected with satisfying matching score. (Further detected proteins can be found in Section S3, Supporting Information) Moreover, we performed western blots to confirm the findings and detect proteins that might still have been below the limit of detection of the MALDI‐TOF‐MS method. 15% w/v polyacrylate gels have been employed for a maximum resolution between these rather small proteins. Neither OmpC, OmpF nor OmpA could be detected on *E. coli* BL21 DE3 Omp8 OMVs, which confirmed the suitability of these OMVs as porin‐less control (Figure S4A,B: Supporting Information). In contrast, a strong OmpF band (Figure S4B: Supporting Information, red band) and fainter OmpC and OmpA bands (Figure S4A: Supporting Information, green or red band, resp.) were found for *E. coli* BL21 DE3 OMVs, confirming this type of OMV to be appropriate to study the impact of porin‐mediated permeation. Only a very faint OmpA band and no band for OmpF and C could be found for OMVs of *E. coli* MG1655, which confirmed earlier findings that trimeric porins tend to be in lower amounts on OMVs.^[^ ^5^ ^]^

Western blot was also performed to investigate the presence of LPS via their component lipid A on the vesicle surface, which could be confirmed for both the BL21 DE3 as well as the BL21 DE3 Omp8 OMVs taking their bacterial lysate as a reference. (Figure S5, Supporting Information)

### 2.3 Preparation of the OMV‐Based In Vitro Model

**Figure 5:**
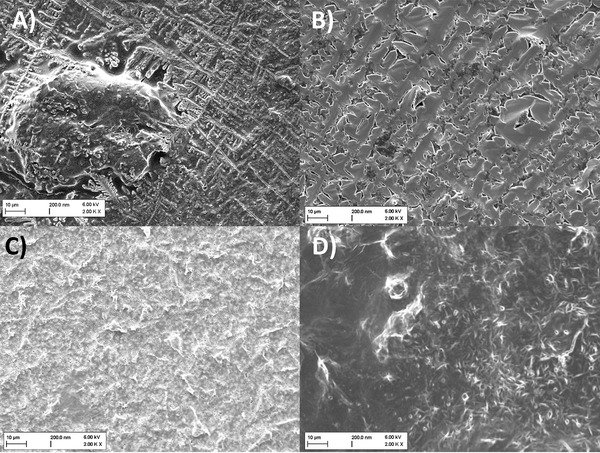
SEM micrographs of various treated filter supports. A) *E. coli* BL21 DE3 OMV‐derived filter coating after three coating steps. B) Filter support after three coating steps with phosphate buffered saline. C) Uncoated filter support. D) Filter support after six coating steps with OMVs from *E. coli* BL21 DE3.

For the coating of filter supports with OMVs, we adapted previously established protocols.^[^ ^18^, ^37^ ^]^ Considering the comparatively low yield of OMVs, the particle concentration was standardized to 10^12^ particles mL^−1^, which could be easily obtained for OMVs from *E. coli* strains BL21 DE3, BL21 DE3 Omp8 as well as for the comparator liposomes. A concentration of 10^12^ particles mL^−1^ can be considered as a rather low concentration for macroscopic coating applications. However, the application of such low concentrated vesicle suspensions likely leads to coatings with advantageous properties for permeation studies, such as reduced membrane retention of permeating compounds and functional porins. Moreover, the drying and fusion process was conducted at 37°C instead of 60°C, but under strong ventilation, allowing to keep a time span of 1 h for each drying cycle. The previously reported freeze‐thaw cycling was omitted, as according to SEM analysis of the obtained membranes, the vesicles had already lost their individuality after the drying step (**Figure** **5**A).

Notably, small crystals originating from the buffer salts stretch across the fused vesicle layer. However, these are clearly different from the pure buffer crystals (Figure 5B) and the surface structure of the uncoated filter. (Figure 5C) Moreover, the OMV coating features amorphous parts, as previously observed for PL coatings.^[^ ^18^ ^]^ Further iterations of the coating process resulted in an enhancement of the amorphous topology. (Figure 5D) Yet, due to the limited OMV yield and in order to facilitate maintaining the functionality of OM proteins, we decided to continue with only three coating cycles.

By looking from above on membranes prepared in this way, one can notice that there are common patterns regardless of which vesicular material has been employed. At the outer zones of the filter support, salt crystals from the phosphate‐buffered saline (PBS) appear. These become smaller and less abundant toward the center of the support (Figure S6, Supporting Information). Close‐up views of the center of the filter support reveal an inhomogeneous and incomplete coating of salt crystals and amorphous matter (Figure S7, Supporting Information).

**Figure 6:**
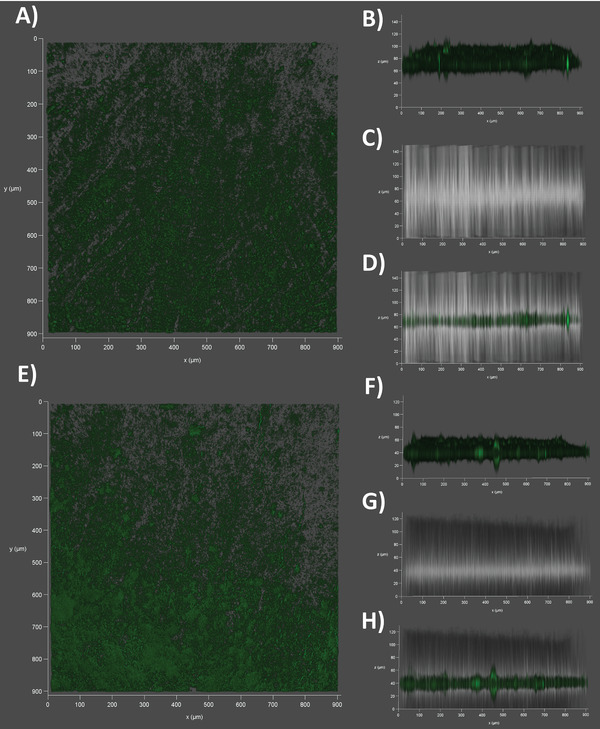
Confocal Laser‐Scanning Microscopy of the outer membrane vesicle (OMV)‐derived membrane model. A) Top view on an NBD‐PE‐stained (green) *E. coli* BL21 DE3 OMV‐coated filter support at dry state. B) Sideview on the same dry membrane using the fluorescence channel or C) the brightfield. D) Superimposition of fluorescence and brightfield channel. E) Top view on the same NBD‐PE‐stained and OMV‐coated filter support at wet state, after incubation for 30 min in PBS (pH7.4). F) Sideview on the membrane using the fluorescence channel or G) the brightfield. H) Superimposition of fluorescence and brightfield channel.

According to CLSM observations between the center and edge of the in vitro membrane model composed of NBD‐PE‐stained OMVs from *E. coli* BL21 DE3, the obtained membrane is slightly inhomogeneous with some non‐fluorescing patches. (**Figure** **6**A,E) The membrane thickness at dry state is about 20 µm (Figure 6B) and superimposing fluorescence and brightfield channel (Figure 6C,D) revealed that most of the PL is located inside the filter support. The same holds true after incubation with PBS for 30 min. (Figure 6G,H) However, the membrane thickness increased to 40 µm. (Figure 6F) CLSM images of the comparator membranes derived from *E. coli* BL21 DE3 Omp8 OMV (Figure S8, Supporting Information), bacteriomimetic liposomes (Figure S9, Supporting Information) and eukaryotic liposomes (Figure S10, Supporting Information) also revealed an inhomogeneous coating. Moreover, the liposome‐based models seem partially more uncoated and thinner. However, after hydration, all membranes tend to increase in size and show a much more confluent coating of the filter support. This tendency of the membrane to become thicker was also confirmed by a quantitative evaluation of the fluorescence signals obtained from the different membrane types. (Figure S11, Supporting Information) Conclusions regarding the actual membrane thickness and coating efficiency must be, however, done with caution, since the NBD‐conjugated phospholipid PE may insert in the different vesicle membranes with different efficiency. Based on the results obtained from CLSM and SEM, it seems likely that throughout all employed types of vesicles, the coating material impregnated partially the pores of the filter membrane, while partially also accumulating at the inner wall of the well.

Further investigation on the physical state of the coating is performed using SAXS. The scattering pattern of OMVs coated on a membrane filter displayed only a faint characteristic peak around 0.075 Å^−1^, which is not observed in the scattering pattern of the empty membrane filter (Figure S12A, Supporting Information). This indicates that only a very small fraction of the entire coating is arranged in an ordered state with a repeat distance of ≈8 nm. It can be concluded that the rich and varied composition of OMVs strongly hinders the formation of ordered structures. Also after incubation in PBS (Figure S12B, Supporting Information), the system remains mainly disordered. Membrane filters coated with bacteriomimetic liposomes, however, showed two very distinct peaks, which indicates a larger number of ordered PLs (Figure S12C, Supporting Information). They are positioned at *q* = 0.113 and 0.135 Å^−1^, which corresponds to repeat distances of 5.6 and 4.7 nm, respectively. Presumably, these represent bilayers of POPE and POPG that form the liposomes. Hydrating this coating led to the merging of both intensity peaks, and, therefore, to the fusion of both structures into a single one (Figure S12D, Supporting Information). All of the discussed scattering peaks did neither appear when a membrane filter was coated with pure PBS buffer nor when the buffer‐coated membrane filter was rehydrated (Figure S12E,F: Supporting Information).

Simultaneous WAXS measurements provided further insights into the structures on the atomic level. In their dry states, the scattering patterns of both OMV‐coated membrane filters (Figure S13A, Supporting Information) and membrane filters coated with bacteriomimetic liposomes (Figure S13C, Supporting Information) do not strongly deviate from that of the empty membrane filter. In both cases, only a few weak and narrow peaks are visible, which are also observed in the scattering pattern of a membrane filter coated with pure PBS buffer (Figure S13E, Supporting Information). Thus, these peaks stem from scattering at small crystals originating from the buffer salts, as was also shown by SEM (Figure 5). In their hydrated states, the scattering pattern of OMVs coated on a membrane filter also did not deviate from that of an empty membrane filter, but the coating consisting of bacteriomimetic liposomes showed a significant increase in scattered intensity at *q* values above 1.5 Å^−1^. This contribution presumably stems from water, which is expected to give a very broad peak in the WAXS region. Therefore, the coating consisting of fused bacteriomimetic liposomes remains hydrated in the duration of the measurements (30 min), whereas no sign of water uptake by OMV‐coatings is observed. Both findings support the observations by CLSM, where OMVs from *E. coli* BL21 DE3 (Figure 6B,F and Figure S11: Supporting Information) did not show much swelling compared to the swelling of bacteriomimetic liposome‐derived membrane (Figure S9B,F and S11, Supporting Information).

The lipid coating of the filter support is highly susceptible to mechanical stress, as can occur during the performance of a transport study. Hence, a 0.5% w/v agarose gel layer was placed on top and a thin film on the bottom of the membrane model. Such low‐concentrated agarose gel has already previously shown no significant separating effect between antibiotics.^[^ ^20^ ^]^ The gel coating of the vesicle‐based models has been investigated by stereomicroscopy and showed throughout all four vesicle types a confluent and concave layer of ≈1 mm minimum thickness (Figure S14, Supporting Information).

### 2.4 Functional Assessment of the Membrane Model

#### 2.4.1 Impact of Agarose Gel and Vesicle Coating on Compound Permeation

**Figure 7:**
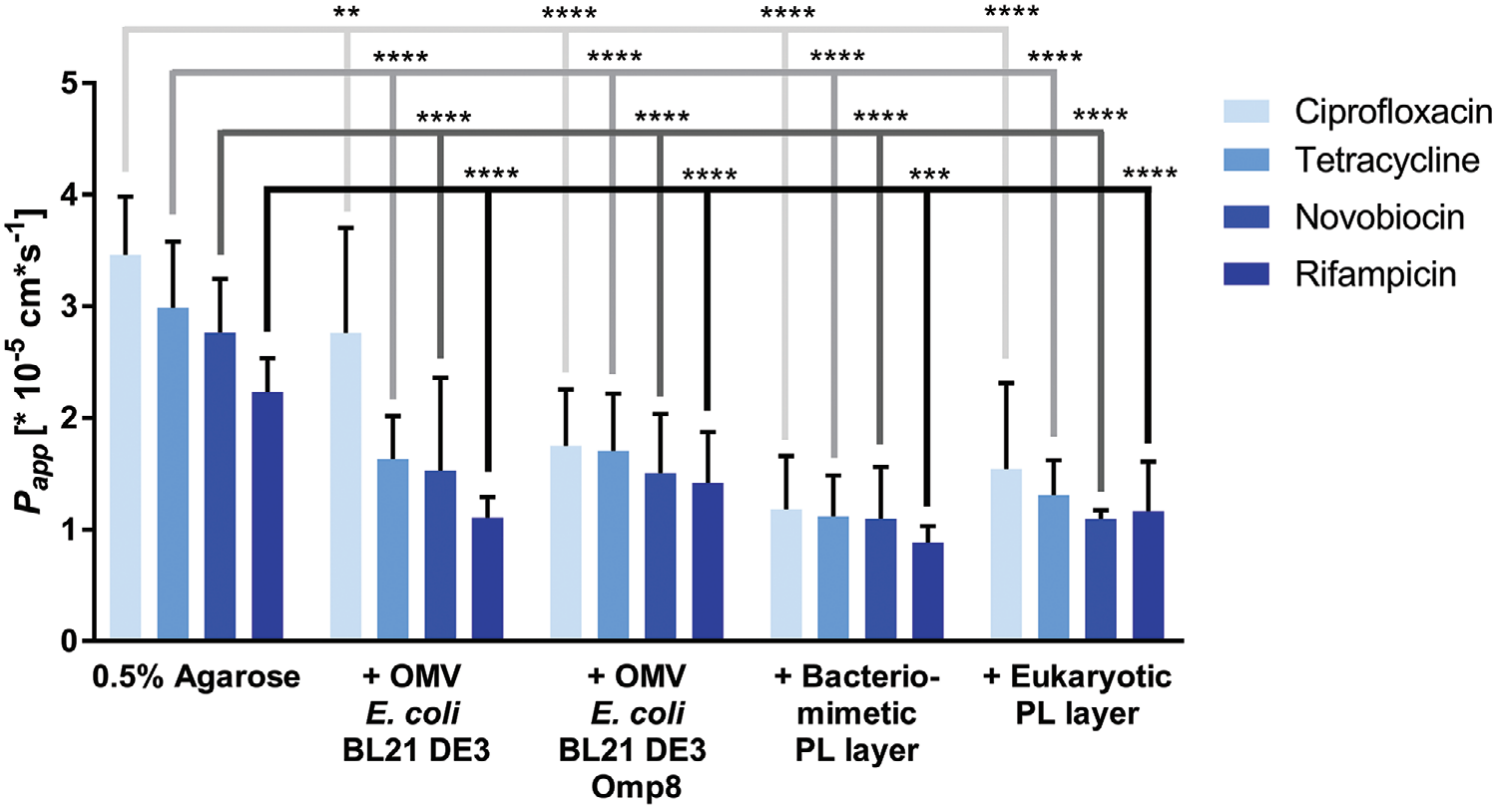
Impact of 0.5% w/v agarose gel on overall compound permeation. By comparing the apparent permeability coefficients (*P*_app_) of four structurally diverse antibiotics (ciprofloxacin, tetracycline, novobiocin and rifampicin) obtained from either pure 0.5% w/v agarose gel coating or combined coating with vesicle suspensions and agarose gel, differences in the permeation velocity become noticeable. The permeation through the pure agarose gel layer is significantly faster than through the respective combinations of agarose gel and vesicle‐based coating. Columns represent mean + SD. *N* ≥ 9 from at least 3 independent experiments. Two‐way ANOVA with Dunnett's multiple comparisons post‐hoc analysis was performed using antibiotic‐specific *P*_app_ values of agarose as a control. **P < 0.01, ***P < 0.001, ****P < 0.0001

For the further investigation of the functionality of the obtained OMV‐based membrane model four types of vesicles were employed: i) OMV of *E. coli* BL21 DE3 featuring the major proteins OmpF and OmpA as well as LPS,^[^ ^40^, ^53^ ^]^ ii) OMV of *E. coli* BL21 DE3 Omp8 being devoid of major porins, iii) bacteriomimetic liposomes with PLs representative for *E. coli* ^[^ ^48^, ^54^ ^]^ and iv) eukaryotic liposomes composed of egg PC. To investigate the compound permeation across these biomaterials, it is necessary to assess if the vesicle coating or the agarose coating is the major permeability‐delimiting layer. According to our findings, the permeability is significantly lower with the vesicle coating compared to pure agarose gel coating. This holds true for small compounds such as ciprofloxacin and tetracycline as well as larger compounds, such as novobiocin and rifampicin. (**Figure** **7**) These results confirm that the antibiotic permeation is mainly delimited by the vesicle‐derived layer.

#### 2.4.2 Biophysical Assessment of the Membrane Model

Natural cell membranes feature typical characteristics, two of which were tested in this study: i) PLs are mobile within the cell membrane and hence are capable of lateral diffusion, and ii) surfactants such as polymyxin B can disrupt the PL bilayer.

**Figure 8:**
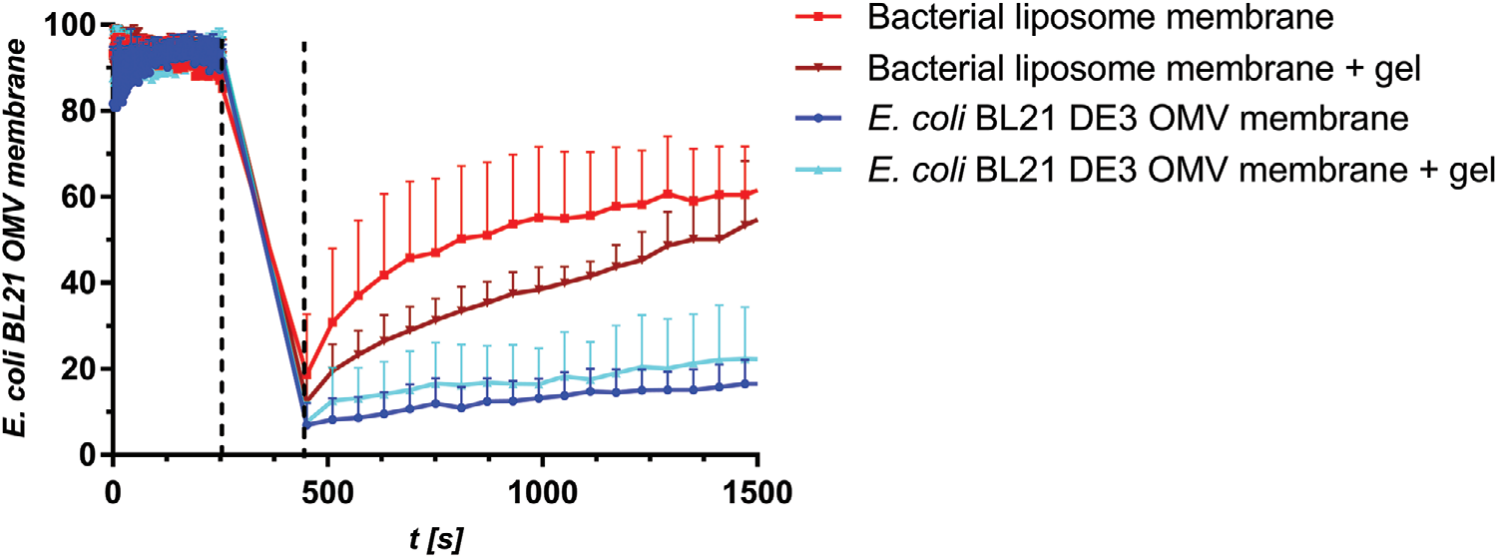
FRAP studies on membrane models derived from OMV of *E. coli* BL21 DE3 or bacterial liposomes. Fluorescence recovery of *E. coli* OMV‐derived membranes is depicted in blue shades while the recovery of liposome‐derived membranes (red dots) is depicted in red shades. Bleaching was conducted for 3 min and started after 255 s as indicated by the dashed lines. Dots represent mean fluorescence from three independent experiments with three regions of interest each.

To investigate the mobility of lipids in our membrane model, fluorescence recovery after photobleaching (FRAP) was investigated on NBD‐PE‐stained membranes derived either from OMVs of *E. coli* BL21 DE3 or bacterial liposomes. Moreover, FRAP was observed either without or with agarose gel coating. (**Figure** **8** and Figure S15: Supporting Information) When looking at the fluorescence‐time course of membranes without agarose gel coating, the liposome‐derived model shows an exponential increase of fluorescence as is typical for PL layers at fluid state. However, the speed of recovery is slower and in the range of minutes rather than seconds.^[^ ^55^ ^]^ In contrast, *E. coli* BL21 DE3 OMV‐derived membranes recover in a much slower and linear manner indicating that among the components of the OMV‐derived membrane the fluid fraction is virtually non‐existent compared to the liposome‐based model (Figure 8). The low fluidity of the OMV coating is in line with the common imagination of the bacterial OM to be highly rigid.^[^ ^56^ ^]^ However, considering the fluidity of OMV‐derived lipid bilayers from either *E. coli* ^[^ ^57^ ^]^ or other Gram‐negative species, ^[^ ^55^ ^]^ the fluidity of this coating appears particularly low, which gives the impression that the functionality of lipids has been dramatically decreased. This is explainable by the additional presence of LPS and proteins among the OMV PLs. When applying the hydrogel, the low recovery profile of the OMV‐based model remains unchanged, whereas the fluorescence recovery of the liposome‐based model becomes hampered, as it follows a linear rather than an exponential trend (Figure 8). It must be mentioned, that conducting FRAP assays and the comparison of their outcome on such types of membranes is challenging, because of their thickness and heterogenicity, compared to plain PL bilayers.

Polymyxin B (PMB) and PMB nonapeptide (PMBN) are classified as cationic antimicrobial peptides, which perturb the membrane, due to the displacement of divalent cations from the LPS layer, enabling other compounds to permeate faster through the membrane. However, none of the membrane‐perturbating agents caused significant changes in the permeability of Fusidic acid and fluorescein, respectively, across OMV‐membrane of *E. coli* BL21 DE3 Omp8 under the tested experimental conditions. (Figure S16A,B: Supporting Information) Interestingly, the lacking ability to permeabilize does not seem to be a feature exclusive for the OMV‐derived models. Besides, the bacteriomimetic liposome‐derived model failed to allow for increased fluorescein permeation after treatment with PMB. On the contrary: fluorescein permeated significantly slower, which is not only in concordance with findings of the change of outer membrane thickness and viscosity under PMB treatment but confirms also the first step of the mechanism of action of PMB, namely the integration of this amphiphilic molecule into the lipid membrane. According to our findings, PMB enriches in the bacteriomimetic liposome‐derived membrane model, making the lipid coating denser and more rigid and consequently less permeable. (Figure S16C, Supporting Information) Because of the thickness, its pronounced swelling in aqueous environment and the pore‐by‐pore distribution of PL the model, however, does not constitute a conventional lipid bilayer to PMB and PMBN that could be perturbed. Considering the overall lack of effect of PMB and PMBN on the OMV‐derived model, one can conclude that the number of PLs is rather low and insufficient in this setup for studying PMB and PMBN interactions.

#### 2.4.3 Comparison of In Vitro Permeation to in Bacterio Accumulation

**Figure 9:**
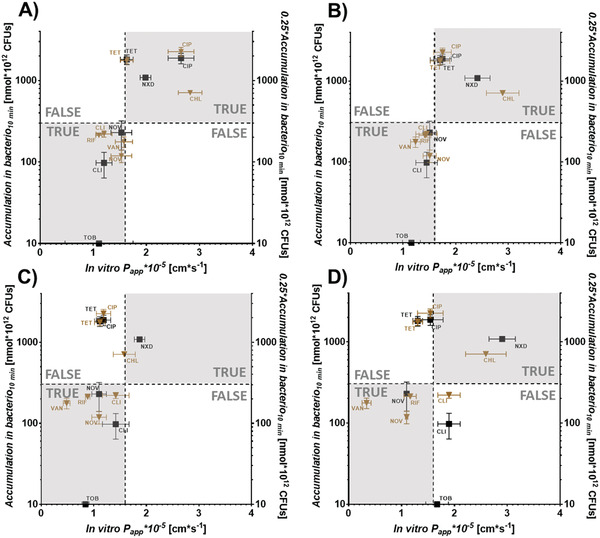
Relationships between in vitro permeability through four vesicle‐derived membrane models and *in bacterio* accumulation in *E. coli* MG1655. Quadrant plots depicting permeability coefficients (*P*_app_) obtained from *E. coli* BL21 DE3 OMV A), *E. coli* BL21 DE3 Omp8 OMV B), bacterial liposomes C) or eukaryotic liposomes D) ‐derived membranes in relation to their reported accumulation. Brown triangles and left *y*‐axis represent accumulation according to Richter et al.,^[^ ^9^ ^]^ while black squares and right *y*‐axis represent accumulation data according to Richter et al.,^[^ ^10^ ^]^ both obtained after 10 min. Horizontal and vertical dashed lines are set arbitrarily at 300 nmol *10^12^ CFUs and 1.6 cm s^−1^, respectively and discriminate high from low accumulating compounds based on Richter et al.^[^ ^9^ ^]^ and Richter et al.^[^ ^20^ ^]^ Gray quadrants demarcate the zone of fast in vitro permeation and high *in bacterio* accumulation (upper right) or slow in vitro permeation and low *in bacterio* accumulation, respectively (lower left). Non‐colored quadrants represent zones of lacking correlation of permeation and accumulation. Points represent mean ± SE. *N* ≥ 9 from at least 3 independent experiments. TRUE and FALSE mark quadrants of true or false predictions, respectively. CHL = chloramphenicol, CIP = ciprofloxacin, CLI = clindamycin, NOV = novobiocin, NXD = nalidixic acid, RIF = rifampicin, TET = tetracycline, TOB = Tobramycin, VAN = vancomycin. Values from Richter et al.^[^ ^20^ ^]^ were multiplied by 0.25 to compensate for the four times higher concentration (200 × 10^−6^ m) compared to Richter et al.^[^ ^9^ ^]^

After characterizing the membrane set‐up on a biophysical level, we investigated the permeability of a set of nine antibiotics across the different vesicle‐derived membrane models in relation to their reported accumulation in *E. coli* MG1655.^[^ ^9^, ^20^ ^]^ The comparison of permeability to intrabacterial accumulation seems ambitious, since other important factors such as efflux and enzymatic degradation also play a role in antibiotic intracellular concentration. In addition, we are aware that the differences in biophysical characteristics observed in the model membranes compared to those in whole cells have a high influence on permeability. Nevertheless, it has been demonstrated previously that the permeability through much simpler biomaterials qualitatively correlated with respective bacterial accumulation data,^[^ ^20^ ^]^ demonstrating the high importance of sufficient antibiotic uptake to overall intrabacterial accumulation. (**Figure** **9**) In this study, we found that in vitro permeability coefficients matched *in bacterio* accumulation best when the permeation model was made of OMV from *E. coli* BL21 DE3 or *E. coli* BL21 DE3 Omp8 (Figure 9A,B), while purely PL‐containing layers as obtained from liposomes failed to adequately match in vitro permeability with *in bacterio* accumulation. Interestingly, the bacteriomimetic model restricted the permeation of virtually all compounds (Figure 9C), while the eukaryotic comparator model allowed clindamycin and nalidixic acid and aminoglycosides to permeate much better than through the OMV‐based models. (Figure 9D) It is important to realize that the selected compounds tetracycline, ciprofloxacin and chloramphenicol are broad spectrum antibiotics and belong to treatment guidelines of Gram‐negative bacterial infections, since they do not only act against *E. coli*, but also more problematic strains such as *Acinetobacter baumannii*, *Enterobacter cloacae, Neisseria gonorrhoeae* and *Salmonella enterica*. Only the more pronounced, resistance of *Pseudomonas aeruginosa* can be either attributed beside low permeability to efflux (tetracycline, chloramphenicol) or enzymatic modification (chloramphenicol).^[^ ^58^, ^59^ ^]^ The fact that the deducted permeation rules (“eNTRy rules”) from *E. coli*‐based accumulation studies yielded anti‐infective compounds with activity against *A. baumannii* and *Klebsiella pneumoniae*, moreover, demonstrates that permeation‐related insights from *E. coli* can still give an added value toward the finding of sufficiently active drugs against more severe bacteria.^[^ ^31^, ^60^, ^61^ ^]^

Remarkably, the porin deficient *E. coli* BL21 DE3 Omp8 OMV‐based model shows similar permeability coefficients to the in vitro model made of OmpF and OmpA‐containing OMV from *E. coli* BL21 DE3. Only the permeability of ciprofloxacin was higher when porin‐containing OMVs of *E. coli* BL21 DE3 were employed. In contrast, purely PL‐based membranes not originating from OMVs lead to a dramatic loss of discriminating power, which becomes even more pronounced the less the PL composition resembles the Gram‐negative OM. The overall permeation pattern of all eight tested compounds indicates that besides porins, other components that are unique to OMVs and the bacterial OM allow for a substantial separation between high‐ and low‐accumulating antibiotics. It is also noteworthy that similar to the accumulation‐related findings by Richter et al. in living wild type and OmpF‐deficient *E. coli*, the permeability of ciprofloxacin dramatically dropped just as seen in Figure 9A,B, while the overall accumulation profile of the compounds did not change.^[^ ^9^ ^]^ As a consequence, testing compounds on the porin‐deficient OMV based model could still yield potential new antibiotic candidates with sufficient uptake.

**Figure 10:**
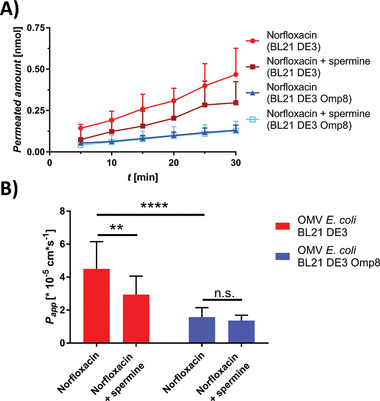
Porin‐blocking experiment on porin‐containing (*E. coli* BL21 DE3 OMV) and porin‐deficient (*E. coli* BL21 DE3 OMV) membrane model. A) Permeation‐time course of norfloxacin in presence and absence of spermine. B) Apparent permeability coefficients (*P*_app_) of norfloxacin in presence and absence of spermine. Points and columns represent mean ± SD. *N* = 12 from three independent experiments. A two‐way ANOVA was performed with Tukey's multiple comparisons post‐hoc test. **P < 0.002, ****P < 0.0001

Since ciprofloxacin permeability was most distinct between the *E. coli* BL21 DE3 and BL21 DE3 Omp8 OMV‐derived membranes and it is assumed to follow porin‐mediated, passive uptake, we wanted to assess if porins could retain functionality after the coating process. Porins have been reported to become blocked in the presence of polyamines such as spermine.^[^ ^62^, ^63^ ^]^ Hence, a porin‐blocking experiment with 140 × 10^−3^ m spermine and 15 × 10^−6^ m norfloxacin was performed on porin‐containing (BL21 DE3 OMV) and porin deficient (BL21 DE3 Omp8 OMV) membrane models (**Figure** **10**), as was done previously in a similar fashion on Enterobacter.^[^ ^63^ ^]^ The permeation of norfloxacin was indeed slightly decelerated when applied together with spermine on the porin‐containing model (Figure 10A), also resulting in significantly different permeability coefficients (Figure 10B). The porin‐deficient model, in contrast, did not only show a significantly decreased permeability for norfloxacin, as could be expected based on the results from ciprofloxacin. It furthermore could not facilitate a significantly slower permeation of norfloxacin in the presence of spermine. This indicates that indeed some functionality of the porins could be maintained.

**Figure 11:**
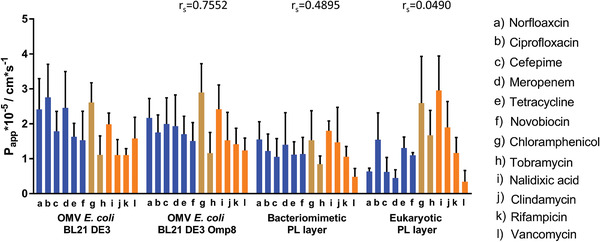
Apparent permeability coefficients (*P*_app_) of eleven antibiotics on four vesicle‐derived membrane models. Antibiotics with expected porin‐facilitated pathway^[^ ^32^, ^63^, ^64^, ^65^, ^66^, ^67^ ^]^ are depicted in blue, while antibiotics with predominantly porin‐independent pathways are depicted in orange.^[^ ^7^, ^15^, ^68^, ^69^, ^70^ ^]^ Antibiotics with various pathways are light brown columns.^[^ ^32^, ^67^, ^70^, ^71^ ^]^ Columns represent mean + SD. *N* ≥ 9 from at least 3 independent experiments. A two‐way ANOVA was performed with Tukey's multiple comparisons post‐hoc analysis (Section S2, Supporting Information).

To obtain a broader view on antibiotic permeability through simpler membrane models compared to the most complex one (i.e., porin‐containing *E. coli* BL21 DE3 OMV‐based), we expanded the panel by the two *β*‐lactam antibiotics meropenem and cefepime. Moreover, we classified the compounds into those with porin‐dependent^[^ ^32^, ^63^, ^64^, ^65^, ^66^, ^67^ ^]^ (blue), porin‐independent^[^ ^7^, ^8^, ^68^, ^69^ ^]^ (orange) and multiple uptake pathways (light brown).^[^ ^32^, ^67^, ^70^, ^71^ ^]^ (**Figure** **11** and Figure S17: Supporting Information) By doing this, we could see gradual changes of the permeability, especially between porin‐dependent and ‐independent compounds, when comparing the vesicle‐derived membranes among each other.

Generally, as found already in the quadrant analysis, *P*_app_‐values of porin‐dependent antibiotics are larger than of antibiotics permeating in a non‐facilitated way when looking at the OMV‐based models. This is also confirmed by their respective permeation‐time course. (Figure S17, Supporting Information) The permeability of fluoroquinolones tends to become lower, the more typical OM features are removed. The lower norfloxacin permeability through BL21 DE3 Omp8‐derived membranes, however, is not as pronounced as in the previous porin‐blocking study (Figure 10). Importantly, the porin‐blocking study was conducted with 15 × 10^−6^ m norfloxacin while the standard permeation assay was conducted with 200 × 10^−6^ m compound concentration as reported earlier.^[^ ^20^ ^]^ Surprisingly the porin‐deficiency of the BL21 DE3 Omp8‐based model did not lead to a significant drop of permeation for the *β*‐lactams, tetracycline and chloramphenicol. This was, however, the case for the liposome‐based models (Section S3, Supporting Information). Here, it is important to note that OMVs of *E. coli* also feature porins other than OmpC, A and F through which uptake can be facilitated. Furthermore, OmpA‐deficient mutants display increased susceptibility to antibiotics since OmpA is associated with membrane integrity. Hence the absence of OmpA in the BL21 DE3 Omp8 OMV‐derived model might have caused a loss of membrane integrity. Moreover, the results suggest that it might be rather the generally larger fraction of hydrophilic components like LPS, proteins and hydrophilic cargo, which make the OMV coating more hydrophilic and hence allow for a better permeation of hydrophilic compounds. The concentration of 200 × 10^−6^ m antibiotic solutions might have also led to partial saturation of porins. By looking at the lipophilic compounds nalidixic acid and rifampicin, no significant difference in permeability could be observed throughout the OMV‐ and the bacteriomimetic liposome‐based model. While rifampicin has also a comparable permeability across the eukaryotic membrane model, the permeability of nalidixic acid increased. The rather large peptide antibiotic vancomycin, which is not expected to be taken via any pathway in a significant manner, still shows the fasted permeation across the BL21 DE3 OMV‐derived membrane model and a gradual decrease of permeability the less the model features components of the Gram‐negative outer membrane. By looking more generally at the model‐specific data sets, one can notice that the eukaryotic membrane model yields the lowest permeabilities for antibiotics that are expected to follow facilitated uptake. In contrast to that, the permeabilities of compounds with non‐facilitated uptake seem less affected by the membrane type. The notion that systematic changes in the vesicle properties affect the overall permeability pattern is also supported by corresponding Spearman rank correlation coefficients (*r*_s_) calculated between the *E. coli* BL21 DE3 OMV‐derived model and the other three comparator models. Also here, *r*_s_ decreases the more typical OM features are missing. The eukaryotic comparator model eventually leads to an almost inverted ranking of compound permeabilities leading to an *r*_s_ of ≈0.

## 3 Conclusion

In this study, we have expanded the concept of phospholipid vesicle‐based membrane permeation assays (PVPAs) to outer membrane vesicles (OMVs) derived from Gram‐negative strains with different porin expression. This approach may provide a novel in vitro tool to predict bacterial bioavailability conveniently and at high throughput. While conventional PVPAs are used to predict oral bioavailability of novel compounds, which mainly follow passive non‐facilitated uptake, outer membrane vesicle‐based permeation assays (OMPAs) allow estimating porin‐facilitated compound permeability across the Gram‐negative bacterial cell wall in a rapid manner without the need to work with living bacteria and in spite of a relatively thick membrane. Apart from optimizing the membrane coating process toward higher homogeneity, the broader implementation of the OMPA for such purposes will depend on suitable methods to increase the yield of OMVs.

## 4 Experimental Section

### Materials

MultiScreen 96‐well filter plates with 0.4 µm PCTE membrane and MultiScreen 96‐well Transport Receiver Plates were obtained from EMD Millipore Corporation (Billerica, Ma, USA). Agarose SERVA (research grade) was obtained from SERVA Electrophoresis GmbH (Heidelberg, Germany). 1‐hexadecanoyl‐2‐(9Z‐octadecenoyl)‐sn‐glycero‐3‐phosphoethanolamine (POPE), 1‐hexadecanoyl‐2‐(9Z‐octadecenoyl)‐ sn‐glycero‐3‐phospho‐(1′‐rac‐glycerol) (sodium salt) (POPG), 1,1′,2,2′‐tetra‐(9Z‐octadecenoyl) cardiolipin (sodium salt) (CL) and 1,2‐dioleoyl‐sn‐glycero‐3‐phosphoethanolamine‐N‐(7‐nitro‐2‐1,3‐benzoxadiazol‐4‐yl) (ammonium salt) (NBD‐PE) were obtained from Avanti Polar Lipids Inc. (Alabaster, AL, USA). Lipoid E80 was kindly donated by Lipoid GmbH (Ludwigshafen, Germany). Tetracycline‐HCl was obtained from chemodex (St. Gallen, Switzerland). Rifampicin was obtained from USBiological (Swampscott, MA, USA). Novobiocin sodium was from Cayman Chemical Company (Ann Arbor, MI, USA) and vancomycin hydrochloride from Alfa Aesar (Thermo Fisher Scientific Inc., Haverhill, MA, USA). Phosphate buffered saline (pH 7.4) was prepared from dissolution of 0.02 m PBS tablets without potassium (Genaxxon Bioscience, Ulm, Germany) in 1 L of Milli‐Q water. Hydrochloric acid and sodium hydroxide solutions (1 m each) were used from Bernd Kraft (Duisburg, Germany). Methanol and Acetonitrile (both HPLC grade) were obtained from VWR Chemicals (VWR International S.A.S., Fontenay‐sous‐Bois, France). Fluoraldehyde (o‐phthaldialdehyde reagent solution) was obtained from Thermo Fisher Scientific (Waltham, MA, USA). Trichloro acetic acid, tobramycin, ciprofloxacin, chloramphenicol, and clindamycin hydrochloride were obtained from Sigma‐Aldrich Co. (St. Louis, MO, USA). Paraffin was obtained from Merck KGaA (Darmstadt, Germany). Polyclonal Anti‐OmpA (origin: rabbit) and OmpC antibodies (origin: rat) were obtained from Antibody Research Corp. (St. Peters, MO, USA). Polyclonal Anti‐OmpF antibodies (origin: rabbit) were obtained from Biorbyt Ltd. (Cambridge, UK). Secondary Alexa Fluor 633‐labelled (origin: Goat) and Alexa Fluor 488‐labelled (origin: chicken) anti‐rabbit antibodies, Alexa Fluor 488‐labelled anti‐rat antibodies (origin: goat) and Alexa Fluor 488‐labelled anti‐mouse antibodies (origin: goat) were obtained from Thermo Fisher Scientific Inc. (Waltham, MA, USA). Monoclonal murine anti‐*E. coli* LPS antibodies [2D7/1] were obtained from Abcam PLC (Cambridge, UK).

### Bacterial Culture

One colony of *Salmonella enterica ssp. Enterica* ATCC 14 028 (strain SL1344), *Pseudomonas aeruginosa* PAO1, *Klebsiella pneumoniae* DSM‐30104*, Escherichia coli* BL21 DE3, BL21 DE3 Omp8 (ΔlamB, OmpF::tn5, ΔOmpA, ΔOmpC) or MG1655, respectively, was transferred into a 100 mL conical flask filled with 20 mL lysogeny broth (LB). After overnight incubation at 37°C, 180 rpm the entire broth was transferred to a 1 L conical flask filled with 280 mL of LB broth, which was cultured over another six nights (37°C, 180 rpm) until isolating the OMV. An additional procedure was chosen for *E. coli* BL21 DE3 and BL21 DE3 Omp8, where after transfer to a 1 L flask the culture was incubated only over one more night.

### Vesicle Isolation

Vesicles were harvested during the death phase (6.95 * 10^10^ CFU mL^−1^, OD_600_ = 4.12). 1 L of bacterial culture was dispensed into Falcon tubes and centrifuged for 15 min (4°C, 9500*g*) using a Hettich Rotina 420 R centrifuge (Andreas Hettich GmbH & Co. KG, Tuttlingen, Germany). Following centrifugation, the supernatant was filtered either through a 0.20 µm Sartorius Minisart NML or 0.45 µm Sartorius Minisart NY syringe filer (Sartorius AG, Göttingen, Germany) The concentration of vesicles was done using either ultracentrifugation or a polyethylene glycol (PEG)‐mediated precipitation method. In the case of ultracentrifugation, the filtrate was equally dispensed into 60 mL ultracentrifugation tubes followed by 2 h centrifugation at 100 000*g* (4°C) using a Beckman Coulter Optima XL‐100K Ultracentrifuge (Beckman Coulter Corp., Brea, CA, USA). In the case of PEG‐precipitation, the filtrate was dispensed into 50 mL Falcon tubes and blended with a 50% m/v PEG 8000‐sultion in a 4:1 ratio. Falcon tubes were kept at 4°C overnight and subsequently centrifuged for 30 min at 16 098*g* (4°C). The supernatant was discarded, and each pellet resuspended in 100 µL of PBS (pH 7.4).

### Preparation of Liposomes

The preparation of bacteriomimetic and mammalian comparator liposomes was done as previously reported.^[^ ^18^ ^]^ In contrast to bacteriomimetic liposomes, mammalian comparator liposomes, were prepared from 233 mg Lipoid E80 (egg phosphatiylcholine), which were dissolved in 5 mL of a blend of chloroform and methanol (3:1). Evaporation, rehydration and extrusion were performed at 50°C.

### Nanoparticle Tracking Analysis (NTA)

Liposomes were diluted 1 in 100 000, while OMVs were diluted 1 in 100 00 in PBS (pH 7.4) before analysis. Each analysis was done in triplicates with 30 s per analysis at 25°C using the Nanosight LM‐10 (Malvern Instruments Ltd., United Kingdom) equipped with a green laser (532 nm). The camera level varied between 12 and 15, while the detection threshold was chosen between 2–5 accordingly. Data processing was performed by Nanosight 3.1 software (Malvern Instruments Ltd., United Kingdom).

### Zetasizing

For zetasizing, liposomes were diluted 1 in 1000 and OMVs were diluted 1 in 100 in PBS (pH 7.4) before analysis using dynamic light scattering to determine the size and laser doppler micro‐electrophoresis for the determination of the zeta potential (Zetasizer Nano ZS, Malvern Instruments, UK).

### SDS‐PAGE

Vesicles of *E. coli* MG1655, BL21 DE3 as well as BL21 DE3 Omp8 were obtained as mentioned above. 20 µL of the obtained samples were given to 5 µL sample buffer (15% v/v deionized water; 50% v/v 0.5 m Tris‐HCl (pH 6.8); 30% v/v glycerol; 10% w/v sodium dodecyl sulfate (SDS); 0.02% w/v bromophenol blue, 5% v/v *β*‐mercaptoethanol), heated to 95°C, and cooled down in ice. A 12% or 15% w/v polyacrylamide resolving gel and 5% stacking gel was prepared. The stacking gel was loaded with 20 µL of sample at 60 V. Gel electrophoresis was performed at 150 V using a Mini‐PROTEAN Tetra handcast system (both Bio‐Rad Laboratories GmbH, Feldkirchen, Germany) and a PageRuler Unstained Protein Ladder (Thermo Fischer Scientific Inc., Waltham, Ma, USA). A Commassie R‐250 solution (1 g L^−1^ Coomassie Brilliant Blue R‐250 in 50% methanol and 50% glacial acetic acid) was employed for protein detection before documenting the results by the Gel Doc EZ System (Bio‐Rad Laboratories GmbH).

### Western Blot

For western blot analysis of outer membrane proteins, SDS‐PAGE was performed as mentioned above, however, protein concentrations were determined by a BCA assay and all concentrations were adjusted to 30 µg mL^−1^. The blotting was done for 1.5 h at 0.1 A under semi dry conditions on a PVDF blotting membrane using a Trans‐Blot SD Semi‐Dry Transfer Cell. Blocking with 5% w/v BSA in tris‐buffered saline with Tween 20 (TBST) was done for 1.5 h during gentle agitation. Incubation with primary antibodies (all diluted 1 in 500) was done at 4°C, overnight during gentle agitation. Incubation with the secondary antibody was done after 1 in 5000 dilution for 1 h at room temperature during gentle agitation. Between each step, the blotting membrane was washed with TBST. Imaging was performed by Sapphire Biomolecular Imager (Azure Biosystems, Inc., Dublin, CA, USA) operating by Sapphire Capture Software [1.0.5.1116]. Alexa 488‐labelled antibodies were detected at an excitation wavelength (*λ*_ex_) of 488 nm and emission wavelength (*λ*_em_) of 518 nm. Alexa 633‐labelled antibodies were detected at *λ*_ex_: 658 and *λ*_em_: 710 nm. pixel size was 200 µm.

For western blot analysis of *E. coli* lipid A, the samples were generated by applying different methods. On the one hand, vesicles were obtained by size exclusion chromatography (SEC) as described previously^[^ ^72^ ^]^ following PEG precipitation. The two fractions with the highest vesicle concentration, as confirmed by both, BCA assay and NTA, were used for a subsequent isolation of lipid A by a modified Bligh and Dyer method (see below).^[^^]^ In addition, fresh liquid cultures of *E. coli* BL21 DE3 and *E. coli* BL21 DE3 Omp8 were used to directly extract lipid A by applying the same Bligh and Dyer method like described as follows (volumes in parentheses indicate volumes from the abovementioned vesicle sample preparation used in this lipid A extraction procedure). Overnight cultures of *E. coli* BL21 DE3 and *E. coli* BL21 DE3 Omp8 were used to inoculate 50 mL of LB medium (at a ratio of 1 to 100). Cells were grown to an OD600 of 0.6 at 37°C. After centrifugation (1000 *g*, 1 h, RT), the supernatant was discarded and the pellet was washed with 50 mL PBS. After centrifugation under the same conditions, the cell pellet was resuspended in 40 mL PBS. To 10 mL of this suspension, 12.5 mL (6.25 mL) of chloroform and 25 mL (12.5 mL) of methanol were added.

The mixture was incubated for one hour at room temperature to ensure complete cell lysis. After subsequent centrifugation (1000 g, 1 h, RT), the supernatant was discarded, and the pellet was washed twice with 50 mL (twice with 25 mL) CHCl3/MeOH/PBS (1:2:0.8). Then, 13.5 mL (6.75 mL) of hydrolysis buffer (50 × 10^−3^ m sodium acetate, 1% SDS, pH 4.5) was added, the mixture was sonicated, and heated in a water bath (100°C, 1 h). After hydrolysis of the oligosaccharide core, 15 mL (7.5 mL) CHCL3 and 15 mL (7.5 mL) MeOH were added and subjected to centrifugation (1000 g, 1 h, RT). The lower phase was collected.

The upper phase was extracted again with 15 mL (7.5 mL) of the lower phase of the two‐phased CHCl_3_/MeOH/H_2_O (2:2:1.8). The lower phase was removed again after centrifugation and combined with the previously removed lower phase. The combined lower phase was washed twice with 20 mL (10 mL) of the upper phase of the two‐phased CHCl3/MeOH/H_2_O (2:2:1.8). The lower phase was collected and immediately evaporated under nitrogen. For western blot analysis, the samples were dissolved in PBS.

Subsequent SDS‐PAGE was performed with a 10% w/v polyacrylamide gel loaded with 10 µL of sample. The blotting was done as mentioned above. Afterward, the membrane was blocked in BSA at room temperature overnight. The primary anti‐*E. coli* LPS antibodies [2D7/1] were diluted 1 in 500 prior to 4 h incubation at room temperature. After washing, the blotting membrane was incubated with secondary Alexafluor 488‐labelled anti‐mouse antibodies for 1.5 h.

### MALDI‐TOF‐MS

Sample preparation: After preparing an SDS gel, bands of interest were cut off and samples were prepared according to Bruker's protocol (In‐gel digest (Coomassie stained) with trypsin; Version 1.0, 06.12.2000). The following steps are in accordance with the protocols provided by Bruker Daltonics. After digestion of the samples, they were dissolved in 10 µL 0.1% trifluoroacetic acid (TFA). An AnchorChip 384 was used as the target type. *α*‐Cyano‐4‐hydroxycinnamic acid (HCCA dissolved in a mixture of 85% acetonitrile (ACN), 15% water, 0.1% TFA and 1 × 10^−3^ m NH_4_H_2_PO4), with a final concentration of 1.4 mg mL^−1^, served as the matrix solution. Subsequently, 0.5 mL of the sample was added to the target, allowed to dry, and then 0.5 mL of the HCCA matrix was added. The Peptide Calibration Standard II (Bruker Daltonics) served as external calibration.

Samples were measured in reflector positive mode, mass‐to‐charge (m/z) range of 700–3500, using Ultraflex MALDI‐TOF/TOF mass spectrometer (Bruker Daltonics) and the flexControl software (Bruker Daltonics, version 3.4). Before a peptide mass fingerprint could be generated, an automated mass list of the monoisotopic peptide signals was generated using flexAnalysis (Bruker Daltonics, version 3.4). The resulting mass fragments were analyzed using Biotools software (Bruker Daltonics, version 3.2), Mascot software (version 2.5.1), licensed in‐house, and the SwissProt database. The settings for the search using the SwissProt database were limited by the taxonomy (*E. coli*), the mass tolerance was set to 100 ppm and one missed cleavage of trypsin was allowed. The fixed modification was assumed to be carbamidomethylation of cysteines and the variable modification was assumed to be oxidation of methionines.

### Cryo‐Transmission Electron Microscopy (cryo‐TEM) of OMVs and liposomes

3 µL of the vesicle suspension were placed onto a S147‐4 holey carbon film (Plano, Germany), followed by blotting to a thin liquid film for 2 s. Afterward, samples were plunged at *T* = 108 K into liquid ethane employing a Gatan (Pleasonton, USA) CP3 Cryo plunge system. Visualization was performed at *T* = 100 K using a JEOL (Akishima, Japan) JEM‐2100 LaB6 TEM operating at an accelerating voltage of 200 kV at low‐dose conditions.

### Scanning Electron Microscopy (SEM) of OMVs

10 µL of vesicles were placed on a TEM‐grid and after a brief rest, the wafer was gently washed 2 times before staining by phosphotungstic acid. The TEM‐grid was mounted on aluminum stubs, using double‐sided adhesive carbon tape and copper grids (Micro to Nano, Netherlands) and let dry overnight at room temperature. Samples were then sputtered with gold using a Quorum Q150R ES sputter‐coater (Gala Instrumente GmbH, Bad Schwalbach, Germany). SEM imaging was facilitated in line integration mode level 5 employing a Zeiss EVO HD15 (Carl Zeiss AG, Jena, Germany) equipped with SmartSEM software (Carl Zeiss AG, Jena, Germany) under an acceleration voltage of 5 kV.

### Coating of Filter Plate

30 µL of the OMV or liposome suspension were given on top of membrane filters of a 96‐well filter plate (MultiScreen 96‐well filter plates with 0.4 µm PCTE membrane). Afterwards, filters were dried at 37°C inside a Memmert UF55 universal oven (fan at 100%). This process was repeated another two times. A six‐fold coating procedure was only followed for SEM‐imaging purposes. For permeation studies, the obtained coatings were covered with 40 µl of 0.5% w/v agarose solution using a direct diplacement pipette. Prior to permeation studies, the bottom of the coated filter was also coated with a thin film of 0.5% w/v agarose using a small brush.

### Small/Wide‐Angle X‐Ray Scattering (SAXS/WAXS)

SAXS experiments were performed using a laboratory‐scale Xeuss 2.0 instrument (Xenoxs SA, Grenoble, France). The X‐ray beam of a copper K_*α*_ source with a wavelength of *λ* = 1.54 Å was focused on the sample with a spot size of 0.25 mm^2^. The scattering signal was measured using a PILATUS 1M photon counting detector (DECTRIS, Baden, Switzerland) at a sample‐to‐detector distance of 550 nm. Simultaneously, WAXS patterns were recorded using a PILATUS 100K‐XEN detector (DECTRIS, Baden, Switzerland), located at a sample‐to‐detector distance of 140 mm and oriented at 36° with respect to the direct X‐ray beam. The combination of both detectors resulted in an accessible range of momentum transfers *q* of 0.02–3.2 Å^−1^, where *q* is defined as *q*  =  4*π*⋅sin(*θ*/2)/*λ*. *θ* is the scattering angle, calibrated using a standard silver behenate sample. All data were azimuthally integrated to obtain *I*(*q*). The sample‐coated membrane filters were placed directly into the X‐ray beam, and measured in transmission mode with an acquisition time of 30 min. The measurements of the dry samples were repeated at least 3 times, and the obtained scattering patterns were averaged. In addition, empty membrane filters were measured as a reference.

### SEM of Coated Filter Plate

Membrane filters were coated with OMVs or liposomes, respectively as previously described. Afterwards they were stripped from the plate and mounted on aluminum stubs, using double‐sided adhesive carbon tape and let dry overnight at room temperature. Samples were then sputtered and SEM was performed as described above using an acceleration voltage of 6 kV.

### CLSM of Membrane

The vesicle staining was carried out with NBD‐PE in a ratio of 100:1 m/m. The required volume of 1 mg mL^−1^ NBD‐PE solution (solvent: chloroform/methanol 1:1 v/v) was given into a microreaction tube. After evaporating the solvent at room temperature, the respective volume of either bacterial liposome or *E. coli* BL21 DE3 OMV solution (10^12^ particles mL^−1^) was added and the diffusion of NBD‐PE into the vesicles was supported by intermitting shaking intervals of 10 min at 37°C and 1000 rpm. The membrane coating was performed as mentioned above. The plate was put on a plastic lid for CLSM observations via Leica TCS SP8 (Leica Microsystems, Wetzlar, Germany). Specifications of the measurement were as follows: the argon laser was used at 20%, the wavelength laser intensity was 12% for the dry sample and the stained membrane was excited at 488 nm. The signal from the dry membrane was detected via PMT2 (511–564 nm) and the gain was 714.3. PMT Trans gain was 290.5 and the pinhole 70.7 µm. A line average of 2 was applied. Resolution was 1024 × 1024 with a physical dimension of 516.67 µm x 516.67 µm. A z‐stack was created over a span of 201.32 nm recording slices of 4.2 µm (48 slices in total). Scan speed was 200 Hz. A 10x objective was employed. The same procedure was conducted for the hydrated membrane, however using a gain of 800.7, a PMT Trans gain of 342.4 and a laser intensity of 11%.

### FRAP

FRAP was investigated using a previously reported protocol with some modifications^[^ ^74^ ^]^ on a Leica TCS SP8. The vesicle staining with NBD‐PE and subsequent membrane‐coating was performed as mentioned above. Prior to FRAP experiments, membranes were incubated for 30 min in PBS (pH 7.4) at 37°C. The buffer was exchanged for fresh PBS (pH 7.4) and FRAP investigations were conducted at 10x magnification at a resolution of 256 × 256 pixel, scan speed: 1800 Hz, pinhole: 1, 50 µm z‐stacks (slice thickness 10 µm) at 37°C with the coated filter wells resting on an inverted plastic lid. Bleaching time was three min (flash frequency: 79 ms) at three regions of interest (circular, size 150 µm^2^, Figure S5: Supporting Information) for each sample using an argon laser (488 nm, laser intensity: 100%). Monitoring of the recovery was performed over 25 min using one scan per minute (laser intensity: 3%, detector: PMT2 (511–564 nm)).

### Stereomicroscopy

Filter wells were coated with the respective sort of vesicles. Such wells were then cut off the plate using a glowing hot blade. Afterward the wells were coated with 0.5% w/v agarose gel as mentioned earlier and embedded in liquid paraffin (55°C). The wells were cross‐sectioned in a cryotome (−20°C, slice thickness: 10 µm) until half of the well remained. The remaining half was mounted on a glass slide. Stereomicroscopic observation was then conducted via a Zeiss Discovery‐V20 stereo microscope featuring an Axiocam 512 color camera and processed with Zen blue software Version 3.2.0.0000 (all by Carl Zeiss AG, Oberkochen, Germany)

### Permeability Studies for In Vitro OMV Membrane‐Based Model – in Bacterio Comparisons

Coated wells were incubated on the apical and basolateral side for 30 min with PBS (pH 7.4) at 37°C while shaken at 180 rpm. After incubation, 230 µL of pre‐warmed 200 × 10^−6^ m antibiotic donor solution (37°C) replaced the PBS in the respective donor wells, while 30 µL were immediately removed and diluted 1:10. The absorbance of these dilutions was measured in a receiver plate using a Tecan Infinite 200 PRO (Tecan Trading AG, Maennedorf, Switzerland) plate reader. (Table S1, Supporting Information) 300 µL of fresh PBS were given into the acceptor wells of the receiver plate followed by absorbance measurements. Donor and acceptor plates were reassembled, incubated, and disassembled after 10, 20, 30, 45, 60, 90, and 120 min to measure the absorbance in the acceptor wells.

In the case of tobramycin, 220 µL donor solution were added on the apical side and samples of 20 µL were drawn and quantified by an o‐phthalaldehyde assay: 200 µL of the commercially available Pierce fluoraldehyde reagent (Thermo Fisher Scientific) were added to each sample and, after an incubation at room temperature, the fluorescence intensity at 470 nm was measured at an excitation wavelength of 360 nm. The removed volume was replaced using fresh PBS (pH 7.4). This protocol was followed for liposome‐based membranes, since no interference of the reagent with membrane components could be observed. For all other quantifications of substance with insufficient *λ*_max_, 240 µL of donor solution were given apically into each donor well. 40 µL were immediately removed and diluted 1 in 5 by with PBS. At all time points, samples of 40 µL were drawn from the basolateral side, diluted 1 in 5 with PBS and measured by LC–MS/MS.

### Membrane Permeabilization Experiments

Experiments were conducted on OMVs of *E. coli* BL21 DE3 Omp8 as stated above with some modifications. Regarding the permeabilization with polymyxin B nonapeptide (PMBN), membranes were incubated for 30 min on the apical and basolateral side either with 103.8 × 10^−6^ m PMBN solution in PBS (pH 7.4) or blank PBS. After 30 min, solutions were removed and the standard protocol was followed employing 200 × 10^−6^ m fusidic acid solution and sampling after 5, 10, 15, 20, 25, 30, 45, and 60 min. The quantification was done via LC–MS/MS.

Regarding the permeabilization with polymyxin B (PMB), membranes were incubated for 30 min either with 83.1 × 10^−6^ m PMB solution in PBS (pH 7.4) or blank PBS. Afterward 240 µL of 10 × 10^−6^ m fluorescein solution in PBS (pH 7.4) was added and 40 µL samples were drawn after 5, 10, 15, 20, 25, 30, 45, and 60 min and diluted 1 in 5 prior to fluorimetric quantification (*λ*_ex_ = 485 nm, *λ*_em_ = 530 nm).

### Porin‐Blocking Experiment

Experiments were performed on OMVs of *E. coli* strains BL21 DE3 and BL21 DE3 Omp8. The procedure followed the standard protocol for permeability studies with some modifications. The membranes were incubated on both sides for 30 min either with 140 × 10^−3^ m spermine solution in PBS (pH7.4) or blank PBS. Afterward, the solutions were removed and 240 µL of 15 × 10^−6^ m norfloxacin solution in PBS containing either 140 × 10^−3^ m spermine or no spermine was added. Samples of 40 µL were taken after 5, 10, 15, 20, 25, and 30 min and analyzed by LC–MS/MS.

### Quantification by LC–MS/MS

An Accela UHPLC System‐Coupled TSQ Quantum Access Max tandem quadrupole mass spectrometer (both from Thermo Fisher Scientific, Waltham, MA, USA) was employed. The entire system was operated via Xcalibur 219 software (Thermo Fisher Scientific, Waltham, MA, USA). The detection of the compounds in the MS happened after heated electrospray ionization (H‐ESI) in positive ion mode. The chromatographic analysis was performed with a binary solvent mixture using optionally acetonitrile + 0.1% formic acid (A), MilliQ‐water + 0.1% formic acid (B), methanol + 0.1% formic acid (C), or ammonium formate buffer (10 × 10^−3^ m, pH 3, D). As for clindamycin, the initial value of 18% A and 72% D was shifted to 30% A and 70% D within 2 min and then kept constant for another 2 min. The gradient run of fusidic acid started with 35% B and 65% C. After 2 min, the values changed to 5% B and 95% C within 1 min. This state was kept constant for 4 min. In the case of norfloxacin, an isocratic binary solvent mixture was used with 18% of A and 82% of D was employed. Vancomycin samples were run for the first minute with 5% A and 95% B before shifting within 1 min to 95% A and 5% B and keeping the values for 3 min.

Tobramycin was analyzed by ion pair chromatography employing Acetonitrile + 0.1% tetrafluoroacetic acid + 0.1% heptafluorobutyric acid + 0.1% pentafluoropropionic acid (E) and MilliQ‐water + 0.1% tetrafluoroacetic acid + 0.1% heptafluorobutyric acid + 0.1% pentafluoropropionic acid (F). The gradient elution started with 80% F and 20% E. After 2.5 min, the ratio shifted to 30% F and 70% E. This state was kept for 1 min. Further LC–MS/MS parameters are summarized in Table S2 (Supporting Information).

### Statistical Analysis

Preprocessing of data (where applicable), data presentation, sample size and statistical methods are detailed in the respective figure captions. To assess significant differences, normal distribution was assumed throughout all data sets and Student's t‐test (Figure S1, Supporting Information) one‐way ANOVA (Figure 3) or two‐way ANOVA (Figures 10, 11 and 7) was applied, followed by posthoc analysis as detailed in the respective figure caption. The Spearman rank correlation coefficient was determined via Excel (Microsoft office 365). The fluorescence signal distribution visualized in Figure S11 (Supporting Information), was obtained from processing the respective z‐stack in imaris software. The following protocol was applied: a surface was created based on the fluorescence signal of the z‐stacks. A section of 400 × 400 pixels (*x*, *y*‐axis) in the upper left side was selected and the surface was calculated (surface detail: 0.5). The automatically suggested threshold was taken and no further adjustments were done. The obtained signal distribution data were exported, the median was set to zero and min, max and 25^th^ as well 75^th^ were adjusted accordingly.

All graphs were designed, and all other statistical analyses were performed via GraphPad Prism software version 7.04 (GraphPad Software, Inc., San Diego, CA, USA).

Raw data obtained from the respective analytical readouts of transport studies were converted to their corresponding concentration via external calibration. A cumulative permeation‐time plot was created using the following formula:

(1) $$m_{accum.}\left(t_{n}\right)=\left(c_{tn}*V_{accept.comp.}\right)-\left(\Sigma m_{t\left(n-x\right)removed}\right)$$

where *m*_accum._(*t*_n_) is the accumulated mass at the timepoint of interest, *c*_tn_ is the concentration at the timepoint of interest, *V*_accept. comp._ is the volume of the acceptor compartment and Σ*m*_t(n‐x)removed_ the total removed mass of compound from all previous timepoints.

The apparent permeability coefficient (*P*_app_) was calculated according to the following formula:

(2) $$P_{app}\;\left(cm*s^{-1}\right)=\frac{J}{c_{0}}$$

where *c*_0_ is the initial donor concentration (µg cm^−^^3^) and *J* (µg cm^−^^2^*s) the compound flux. *J* is calculated by dividing the slope obtained from a linear cumulative permeation‐time plot, at which the drug concentration did not yet exceed 10% of the donor concentration by the surface area of the filter support.

## Conflict of Interest

The authors declare no conflict of interest.

## Supporting information

Supporting Information

## Data Availability

The data that support the findings of this study are available from the corresponding author upon reasonable request.
