# Supplementary material for: An Outer Membrane Vesicle‐Based Permeation Assay (OMPA) for Assessing Bacterial Bioavailability
Source: Adv Healthc Mater. 2021 Oct 24;11(5):2101180. doi: 10.1002/adhm.202101180 (PMC11468809; doi:10.1002/adhm.202101180)
Supplement: Supplementary file 1 — Supporting Information [file ADHM-11-2101180-s001.pdf]

WILEY-VCH

© 2021 Wiley-VCH GmbH

ADVANCED HEALTHCARE MATERIALS

Supporting Information

for *Adv. Healthcare Mater.*, DOI: 10.1002/adhm.202101180

An Outer Membrane Vesicle-based Permeation Assay (OMPA) for Assessing Bacterial Bioavailability

*Robert Richter, Mohamed A. M. Kamal, Marcus Koch, Bart-Jan Niebuur, Anna-Lena Huber, Adriely Goes, Carsten Volz, Julia Vergalli, Tobias Kraus, Rolf Müller, Nicole Schneider-Daum, Gregor Fuhrmann, Jean-Marie Pagès, Claus-Michael Lehr**

Supporting Information

An Outer Membrane Vesicle-based Permeation Assay (OMPA) for Assessing Bacterial Bioavailability

*Robert Richter, Mohamed A. M. Kamal, Marcus Koch, Bart-Jan Niebuur, Anna-Lena Huber, Adriely Goes, Carsten Volz, Julia Vergalli, Tobias Kraus, Rolf Müller, Nicole Schneider-Daum, Gregor Fuhrmann, Jean-Marie Pagès, Claus-Michael Lehr**

2. Results

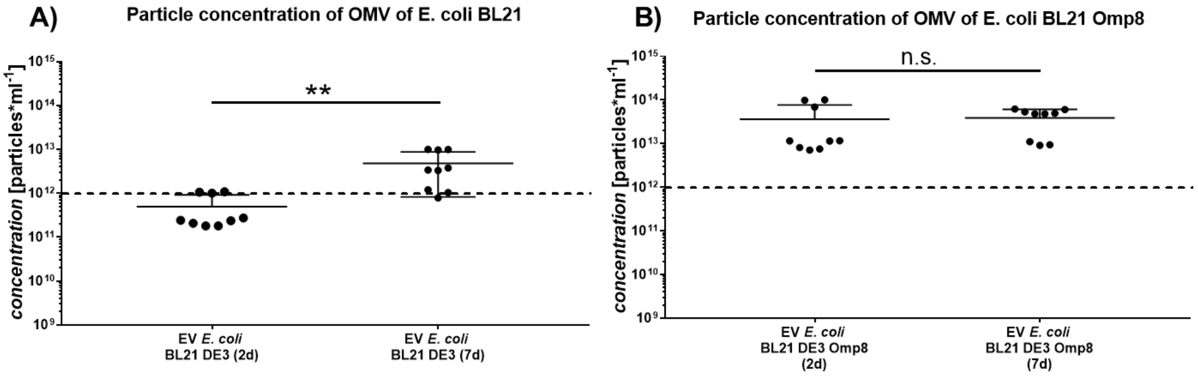

Figure S1. Vesicle yields of (A) *E. coli* BL21 DE3 and (B) *E. coli* BL21 Omp8 after two or seven days of culture. While the vesicle yield from *E. coli* BL21 DE3 could be increased by extending the culture time, *E. coli* BL21 Omp8 did not show significant time-dependent differences. PEG 8000 (blue lines) resulted in a higher yield of OMV than ultracentrifugation (UC), while the pore size of the filter altered the size distribution but not the overall yield. Each line represents mean size distribution. N ≥ 9 from 3 independent experiments. A two-tailed unpaired student's t-test was performed. **P = 0.0052

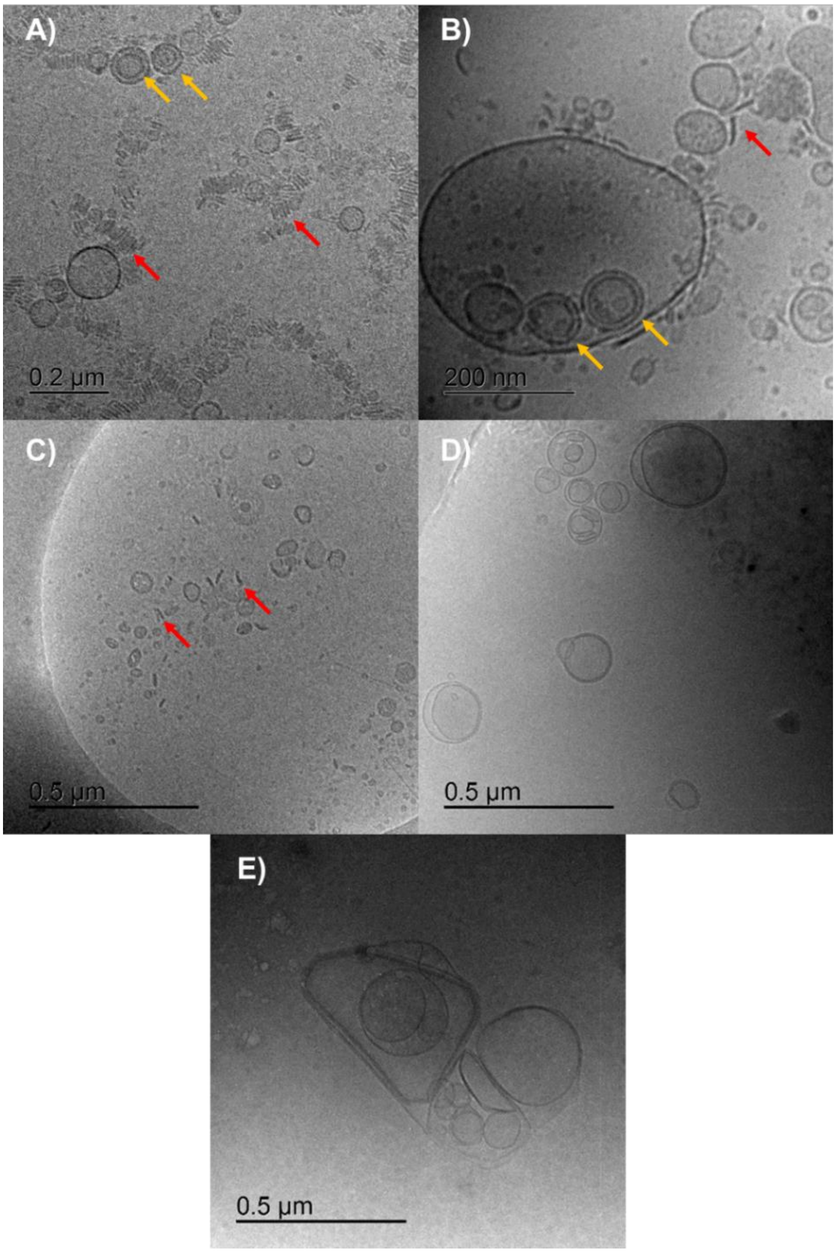

Figure S2. Cryo-TEM micrographs of vesicle structures at diluted state (10^{12} particles/ml). For outer membrane vesicles (OMVs) obtained by PEG precipitation from (A) *E. coli* BL21 DE3 as well as (B) *E. coli* BL21 DE3 Omp8, multilamellar vesicles were observed in low quantity (yellow arrows). (C) OMV suspensions of *E. coli* MG1655 obtained by ultracentrifugation also showed linear lamellar structures as in (A) and (B) (red arrows). In contrast to (D) eukaryotic comparator liposomes composed of egg phosphatidylcholine, (E) bacterial liposomes made of POPE, POPG and cardiolipin have a less spherical and more rigid appearance at a concentration of 10^{12} vesicles/mL.

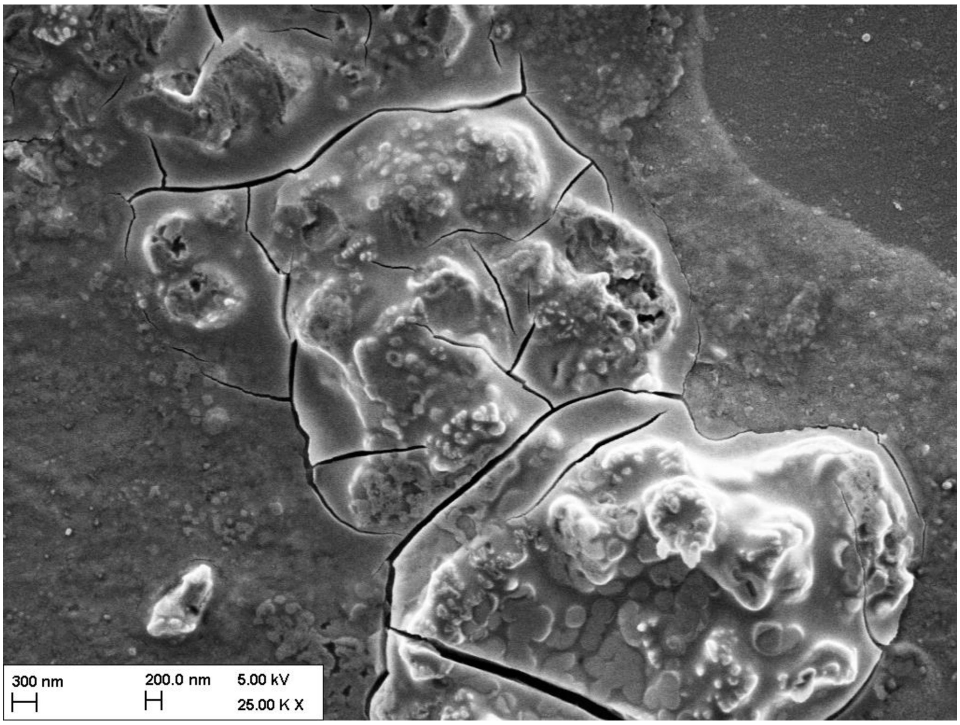

Figure S3. Scanning electron micrograph of dried outer membrane vesicles from *E. coli* BL21 DE3. Tightly packed vesicles tend to fuse in the course of the drying process.

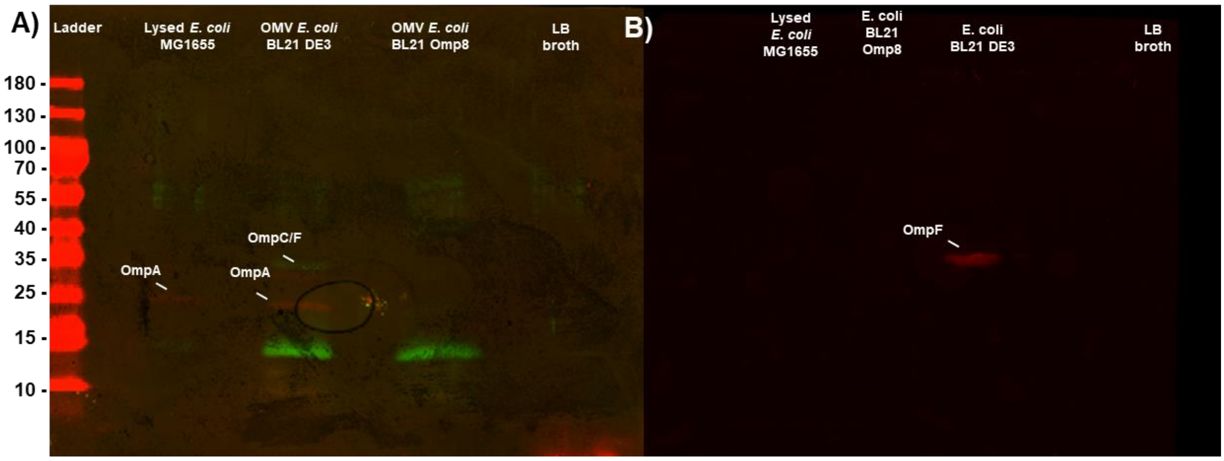

Figure S4. Detection of various porins on outer membrane vesicles of *E. coli* BL21 DE3, *E. coli* BL21 DE3 Omp8 and *E. coli* MG1655 by western blot. Western blot analysis for the detection of (A) OmpC and OmpA as well as (B) OmpF. No OM proteins were detected on OMVs from strain BL21 DE3 Omp8. In contrast, faint bands were detected for OmpC and OmpA and a strong band was found for OmpF on OMVs from the Bl21 strain. Due to the homology of OmpF and OmpC, primary antibodies against OmpC may have bound to OmpF, giving a false positive result. Bacterial lysate only allowed for the detection of OmpA.

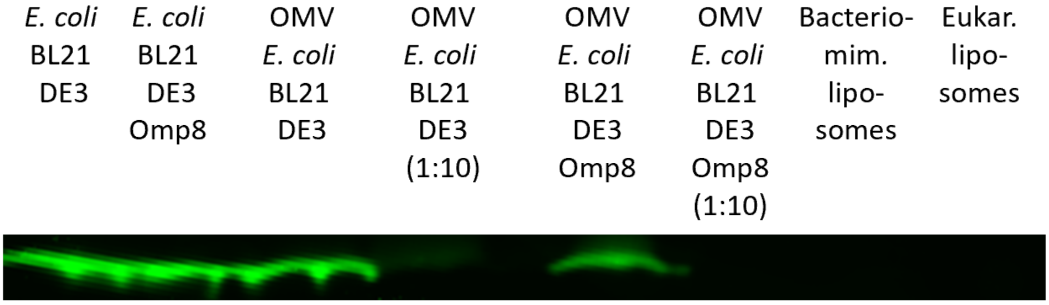

Figure S5. Western blot for the detection of lipid A. Incubation with a fluorescently labelled secondary antibody reveals characteristic bands for the parental *E. coli* BL21 DE3 and BL21 DE3 Omp8 as well as for the outer membrane vesicles.

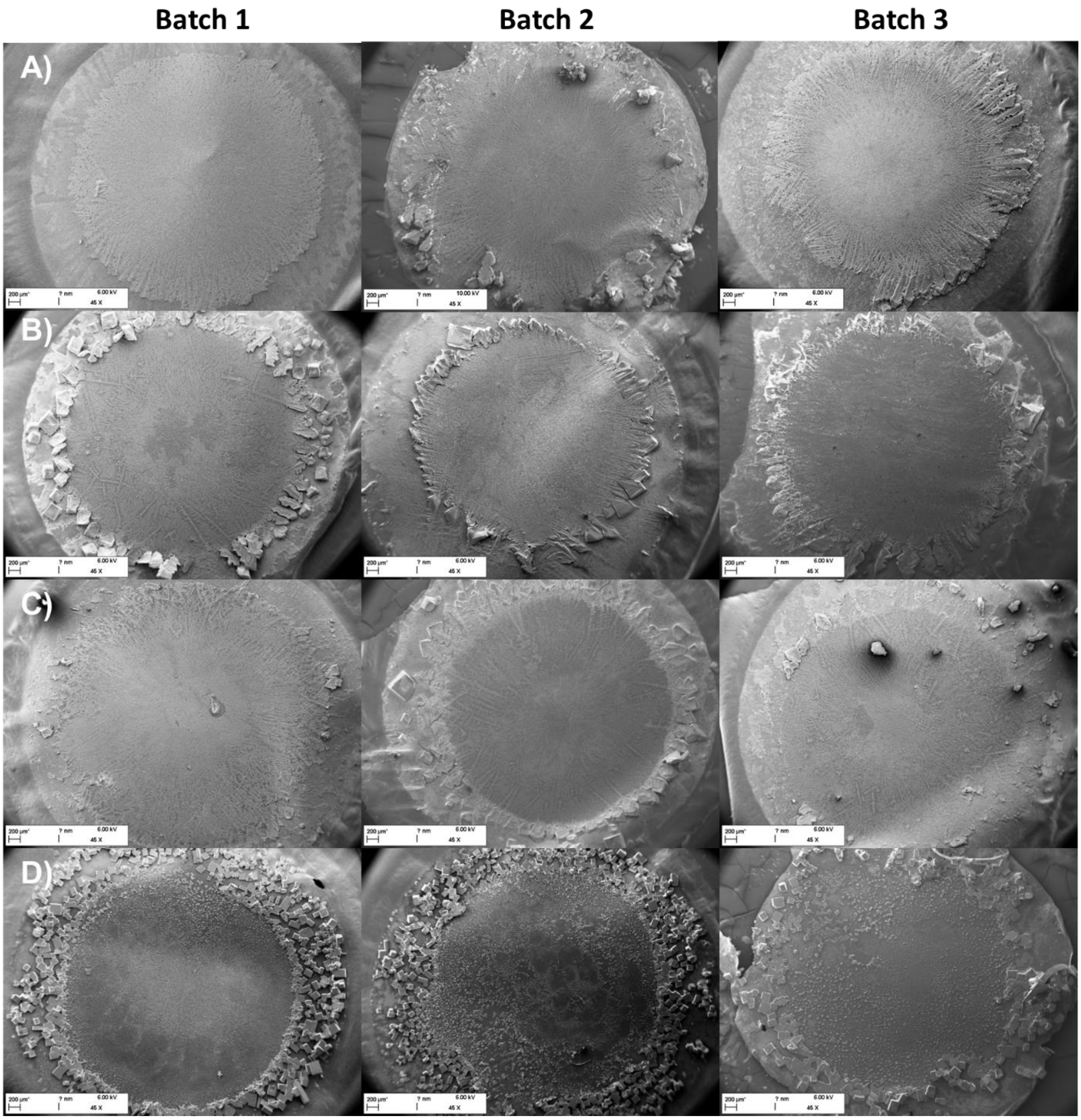

Figure S6. Scanning electron micrographs with complete top view on three batches of membranes. A) Membranes coated with *E. coli* BL21 DE3 outer membrane vesicles (OMV). B) Membranes coated with *E. coli* BL21 DE3 Omp8 OMV. C) Membranes coated with bacteriomimetic liposomes. D) Membranes coated with eukaryotic comparator liposomes.

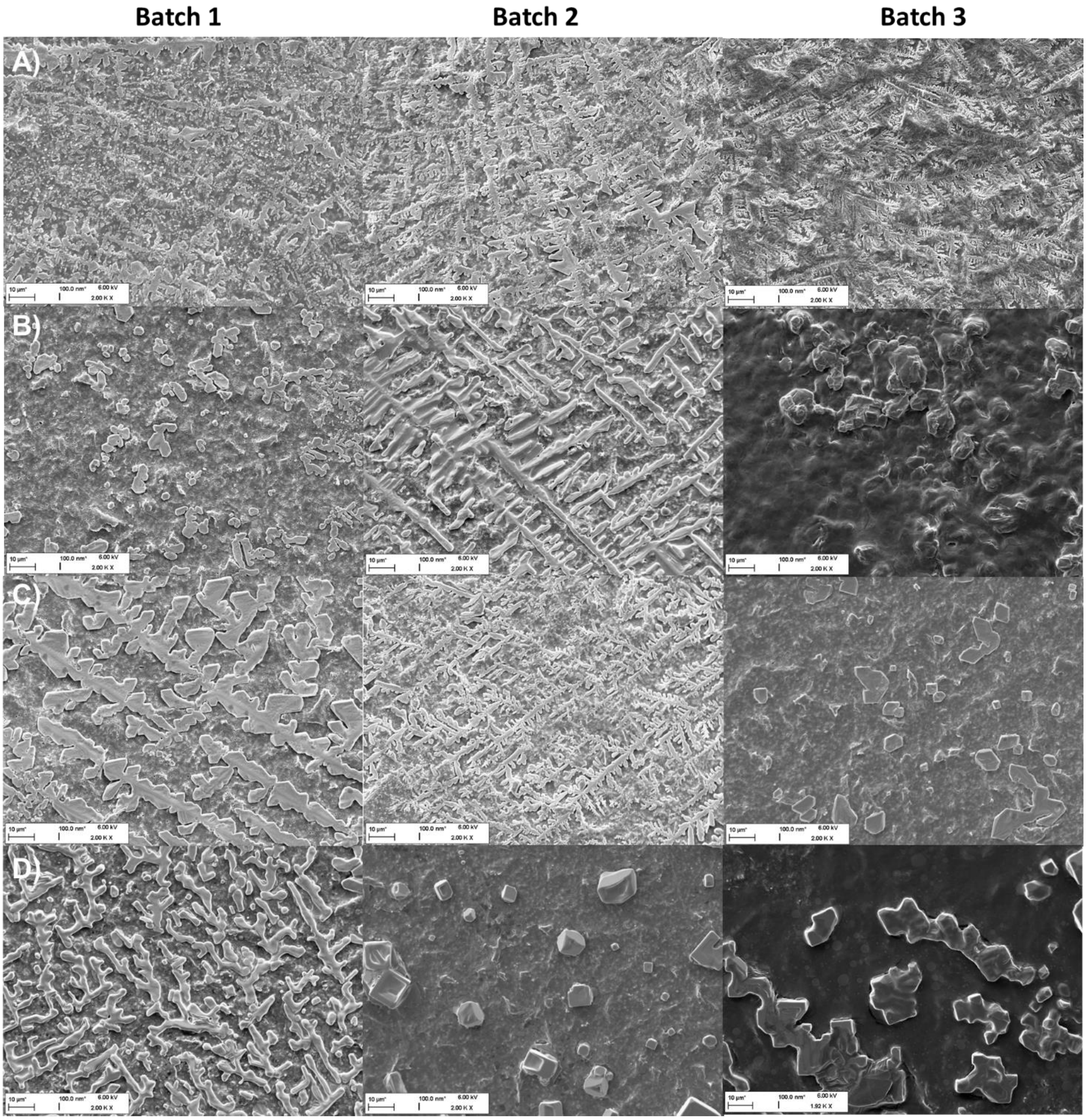

Figure S7. Scanning electron micrographs of the membrane center of three batches of membranes. A) Membranes coated with E. coli BL21 DE3 outer membrane vesicles (OMV). B) Membranes coated with E. coli BL21 DE3 Omp8 OMV. C) Membranes coated with bacteriomimetic liposomes. D) Membranes coated with eukaryotic comparator liposomes.

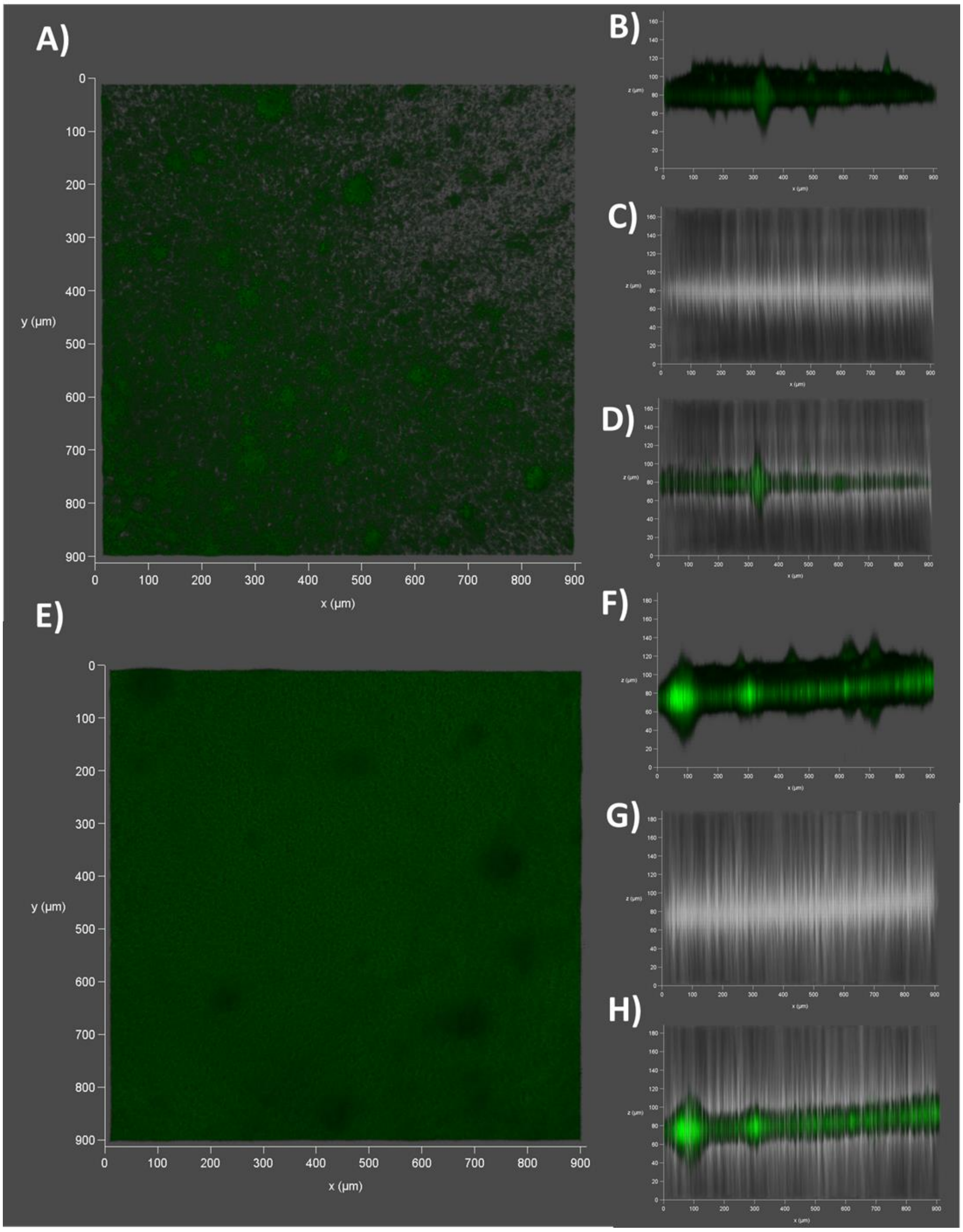

Figure S8. **Confocal Laser-Scanning Microscopy of the *E. coli* BL21 DE3 Omp8 outer membrane vesicle (OMV)-derived membrane model.** (A) Top view on an NBD-PE-stained (green) OMV-coated filter support at dry state. (B) Sideview on the same dry membrane using the fluorescence channel or (C) the brightfield. (D) Superimposition of fluorescence and brightfield channel. (E) Top view on the same NBD-PE-stained and OMV-coated filter support at wet state, after incubation for 30 min in PBS (pH7.4). (F) Sideview on the

membrane using the fluorescence channel or (G) the brightfield. (H) Superimposition of fluorescence and brightfield channel.

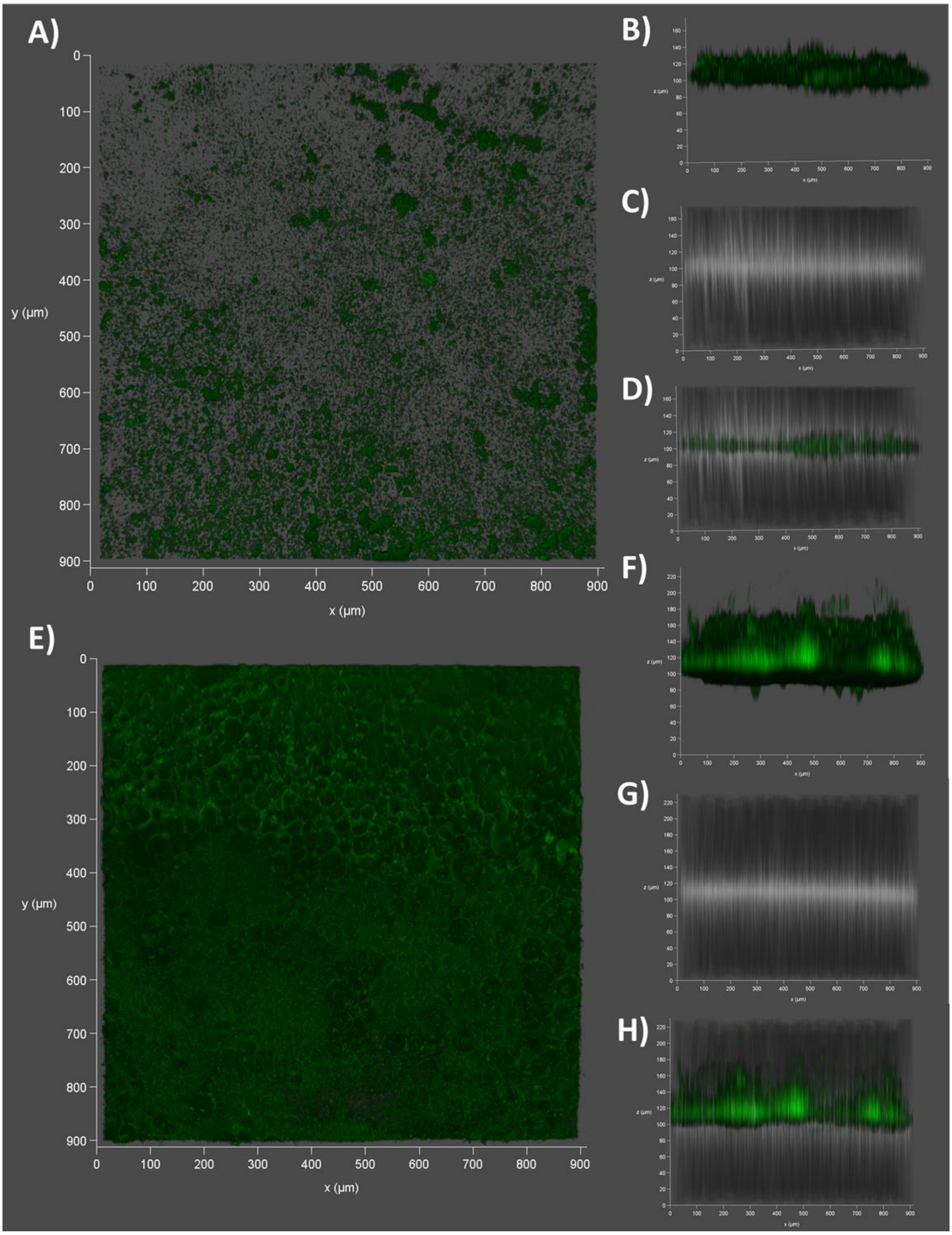

Figure S9. Confocal Laser-Scanning Microscopy of the bacteriomimetic liposome-derived membrane model. (A) Top view on an NBD-PE-stained (green) liposome-coated filter support at dry state. (B) Sideview on the same dry membrane using the fluorescence channel or (C) the brightfield. (D) Superimposition of fluorescence and brightfield channel. (E) Top view on the same NBD-PE-stained and liposome-coated filter support at wet state, after incubation for 30 min in PBS (pH7.4). (F) Sideview on the membrane using the fluorescence channel or (G) the brightfield. (H) Superimposition of fluorescence and brightfield channel.

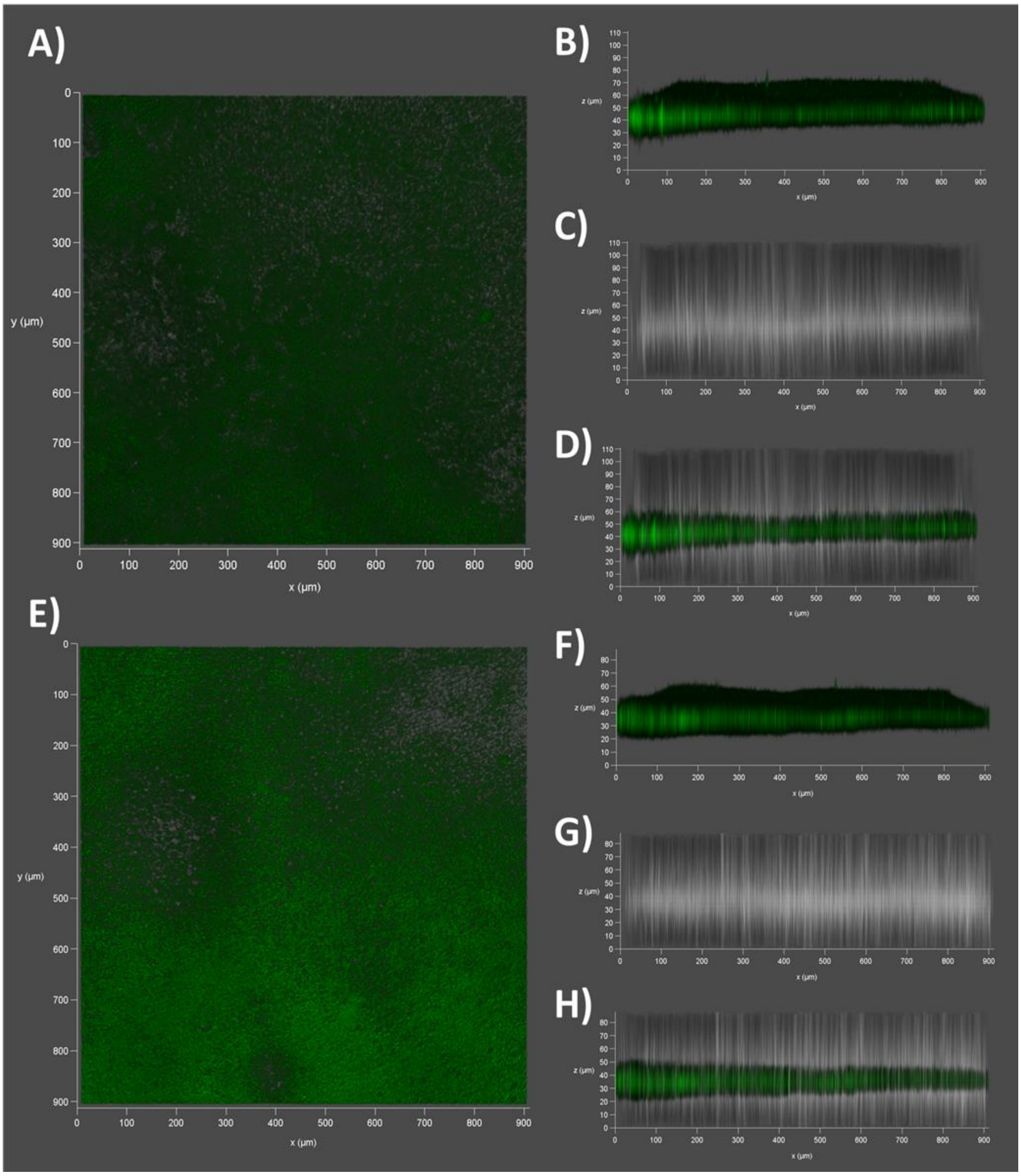

Figure S10. Confocal Laser-Scanning Microscopy of the Eukaryotic comparator liposome-derived membrane model. (A) Top view on an NBD-PE-stained (green) liposome-coated filter support at dry state. (B) Sideview on the same dry membrane using the fluorescence channel or (C) the brightfield. (D) Superimposition of fluorescence and brightfield channel. (E) Top view on the same NBD-PE-stained and liposome-coated filter support at wet state, after incubation for 30 min in PBS (pH7.4). (F) Sideview on the membrane using the fluorescence channel or (G) the brightfield. (H) Superimposition of fluorescence and brightfield channel.

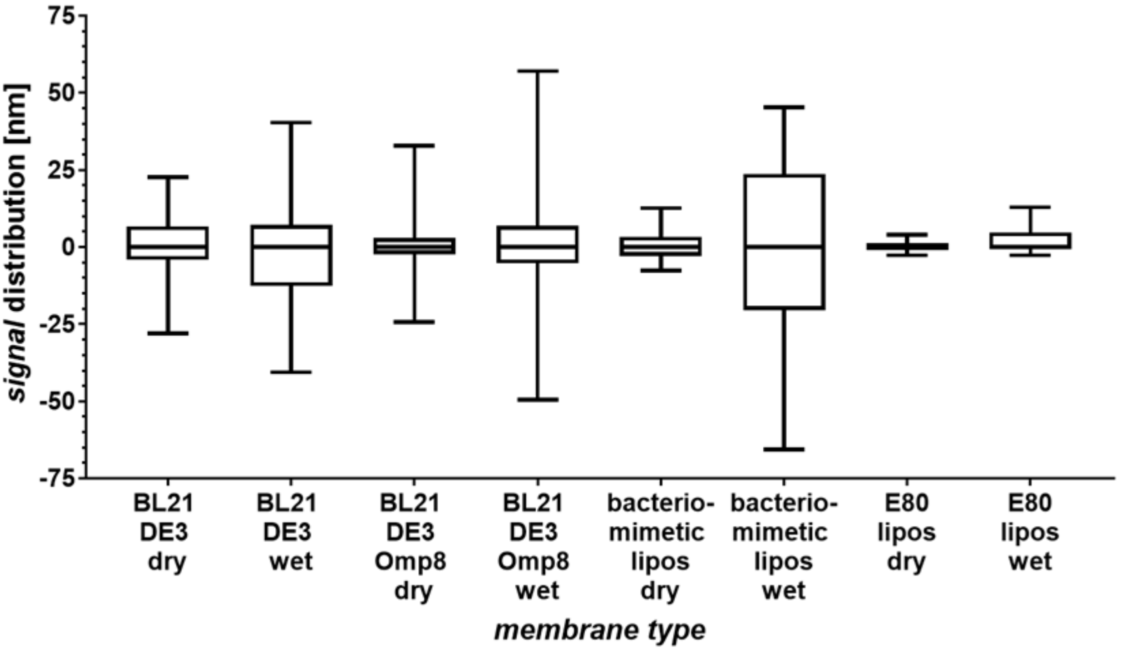

Figure S11. Box-and-whiskers plot of the fluorescence signal obtained from CLSM z-stack analyses. Whiskers represent the minimum and maximum at which a fluorescence signal could be detected. The box is limited by 25th and 75th percentile of the fluorescence distribution data und segmented by the median. For each membrane type data were obtained from three membranes, each one from a different batch.

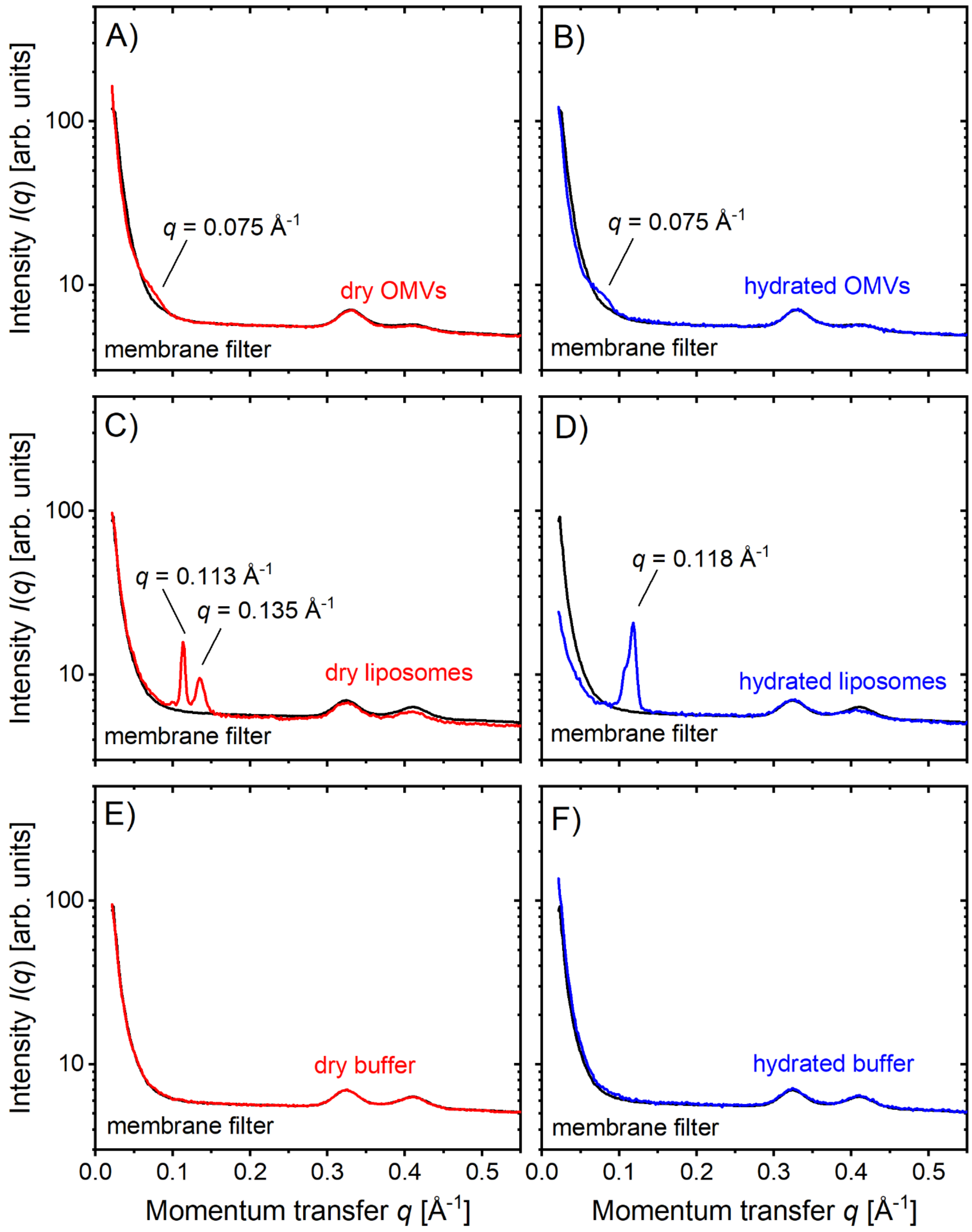

Figure S 12. Small-angle X-ray scattering patterns of dry OMVs (A), hydrated OMVs (B), dry bacteriomimetic liposomes (C), hydrated bacteriomimetic liposomes (D), dried PBS buffer (F) and rehydrated PBS buffer (F). Red curves: dry coatings, blue curves: hydrated coatings, black curves: empty membrane filter.

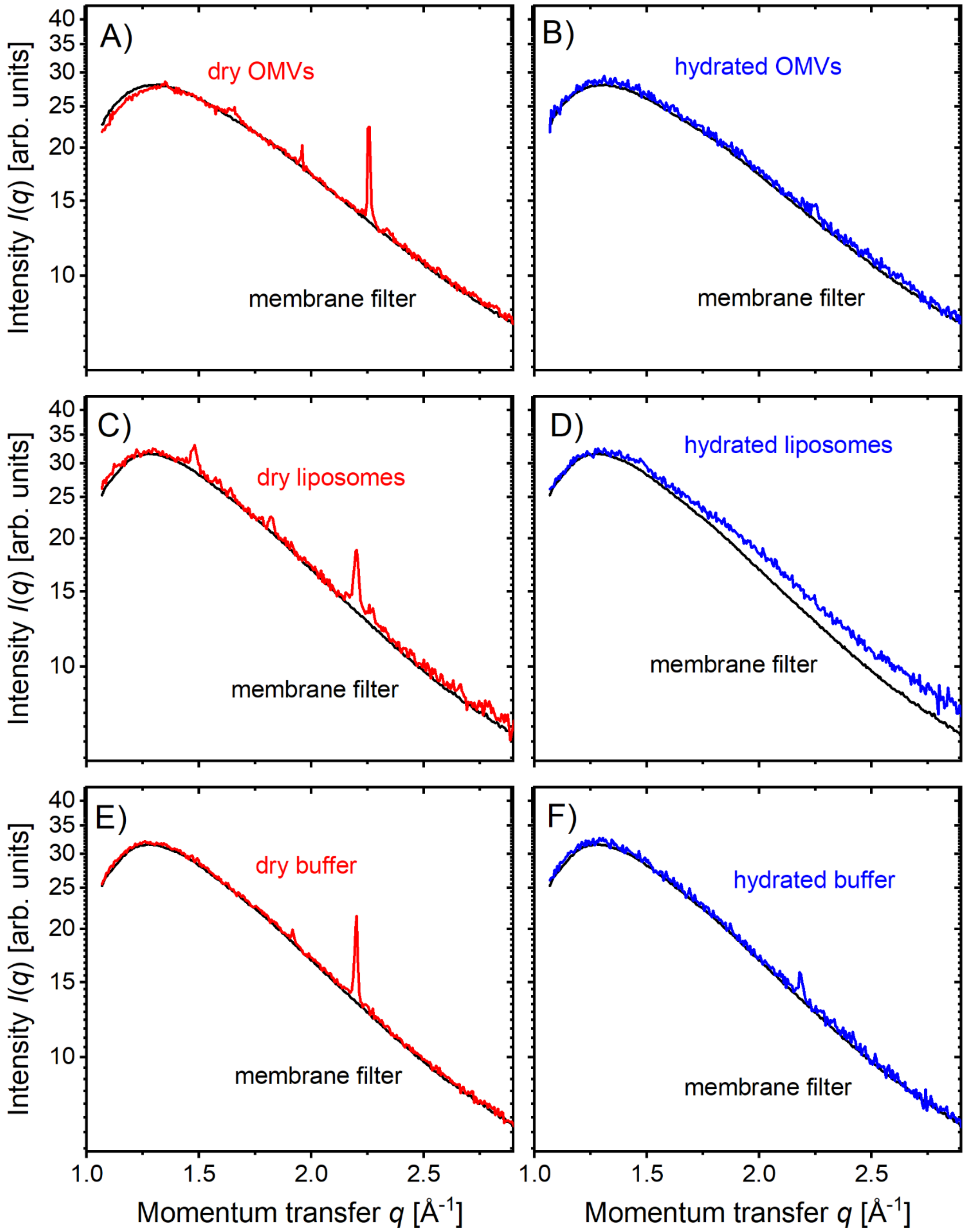

Figure S 13. Wide-angle X-ray scattering patterns of dry OMVs (A), hydrated OMVs (B), dry bacteriomimetic liposomes (C), hydrated bacteriomimetic liposomes (D), dried PBS buffer (F) and rehydrated PBS buffer (F). Red curves: dry coatings, blue curves: hydrated coatings, black curves: empty membrane filter.

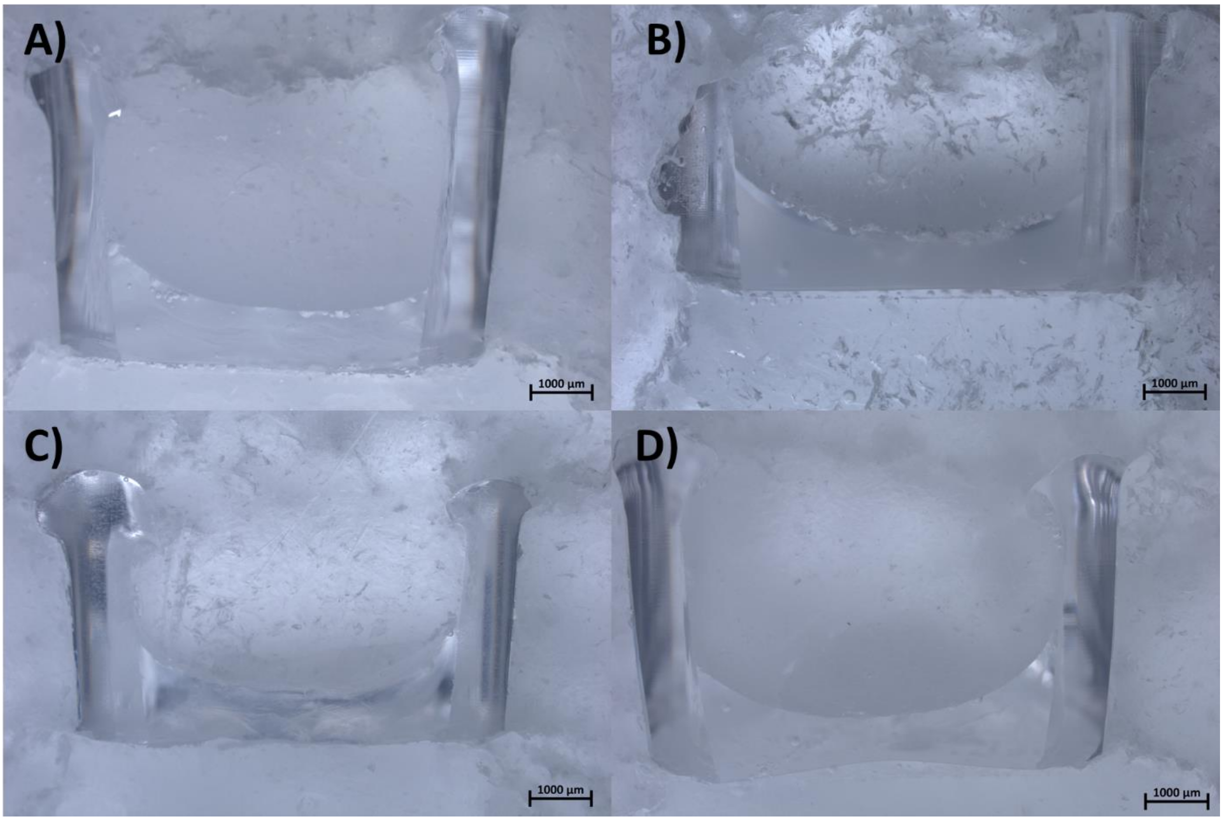

Figure S14. **Stereomicrographs of the vesicle-based models after 0.5% (w/v) agarose gel-coating.** Models derived from OMVs from E. coli BL21 DE3 (A) or from E. coli BL21 DE3 Omp8 (B) as well as from derived from bacteriomimetic liposomes (C) or eukaryotic comparator liposomes (D) are shown as cross-section embedded in paraffine.

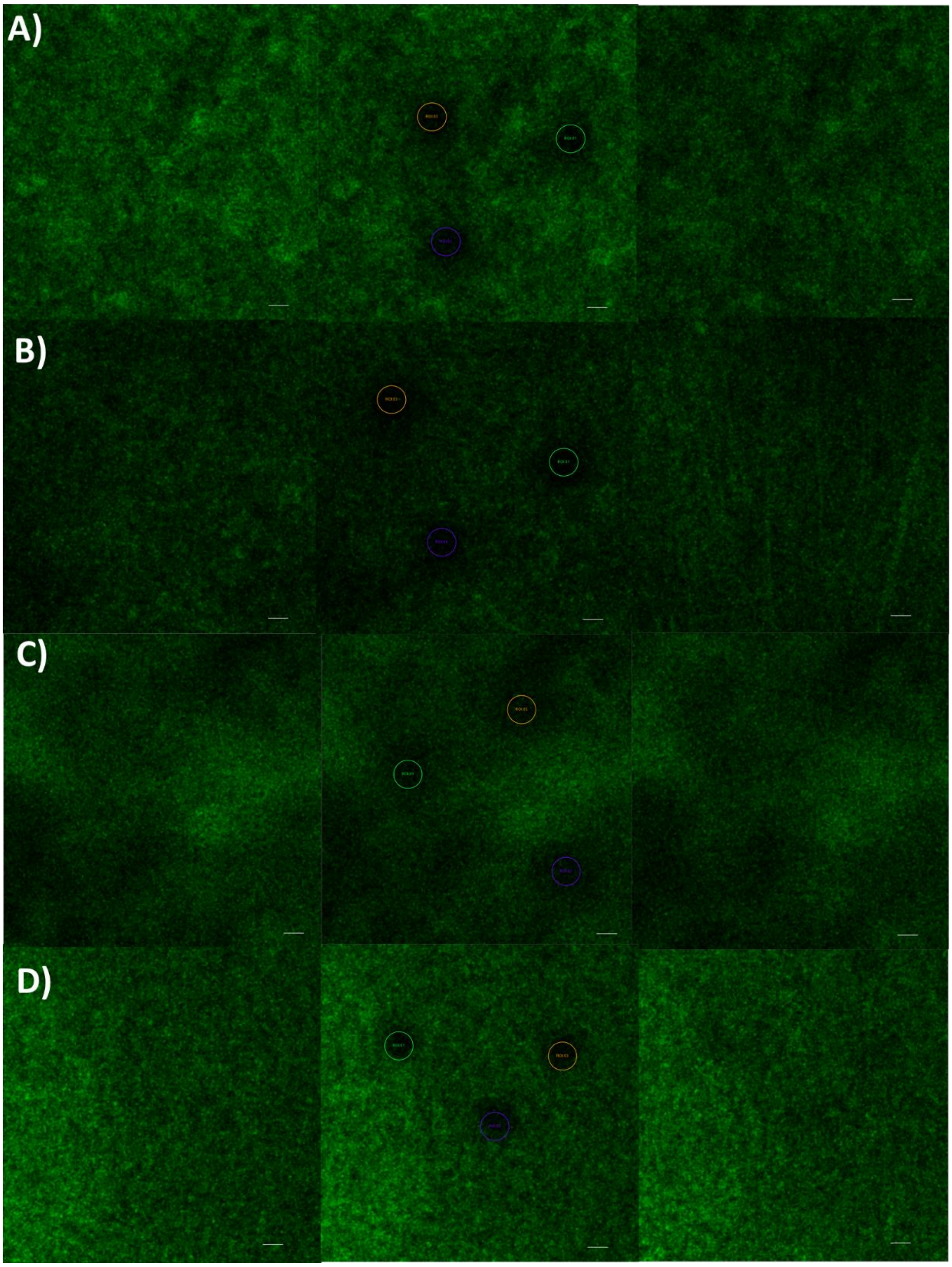

Figure S15. Fluorescence of membrane before bleaching (left), directly after bleaching (middle) and after 25 min recovery (right). A) OMV-derived membrane without agarose gel layer. B) OMV-derived membrane with agarose gel layer. C) Bacterial liposome-derived membrane without agarose layer. D) Bacterial liposome-derived membrane with agarose

layer. All membranes were stained with NBD-PE. Magnification: 10x; scalebar represents 10 µm and colored circles represent bleached regions of interest.

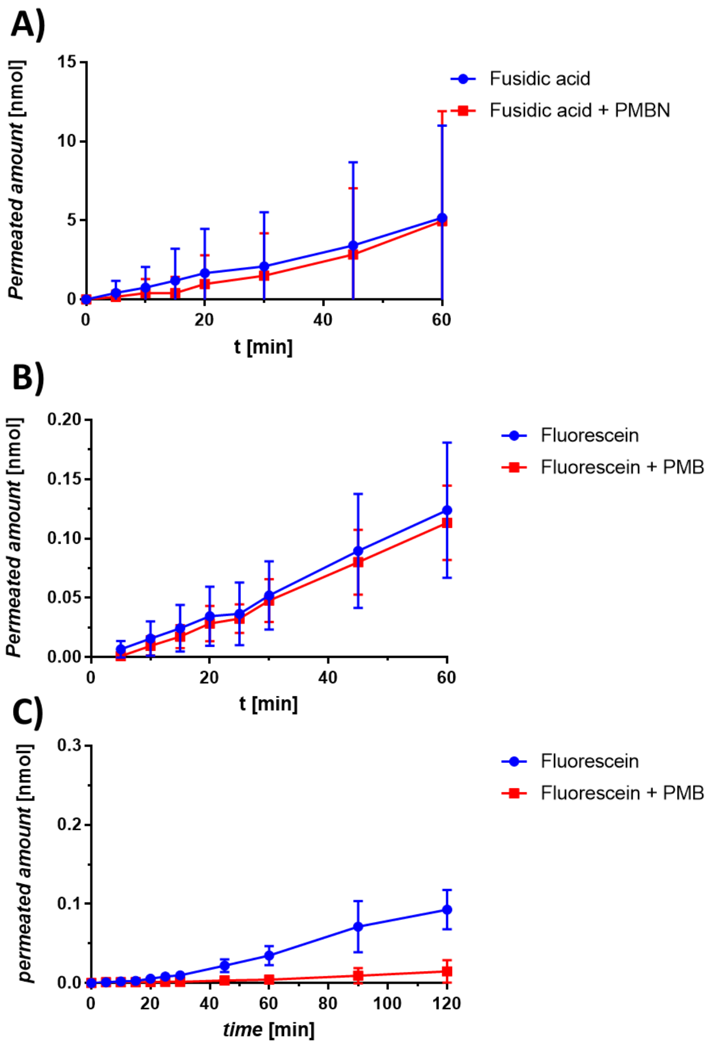

Figure S16. Permeabilization experiments on membrane model derived from OMVs of *E. coli* BL21 DE3 Omp8 and bacteriomimetic liposomes. (A) Fusidic acid permeation following either 30 min incubation of an OMV BL21 DE3 Omp8-derived membranes with polymyxin B nonapeptide (PMBN) or with PBS (pH7.4), respectively. (B) Fluorescein permeation following incubation of an OMV BL21 DE3 Omp8-derived membranes with polymyxin B (PMB, red line and dots) or PBS (blue line and dots), respectively. (C Fluorescein permeation following incubation of bacteriomimetic liposome-derived membranes with polymyxin B (PMB, red line and dots) or PBS (blue line and dots),

respectively. Points and columns represent mean ± SD: $N \geq 9$ replicates from 3 independent experiments.

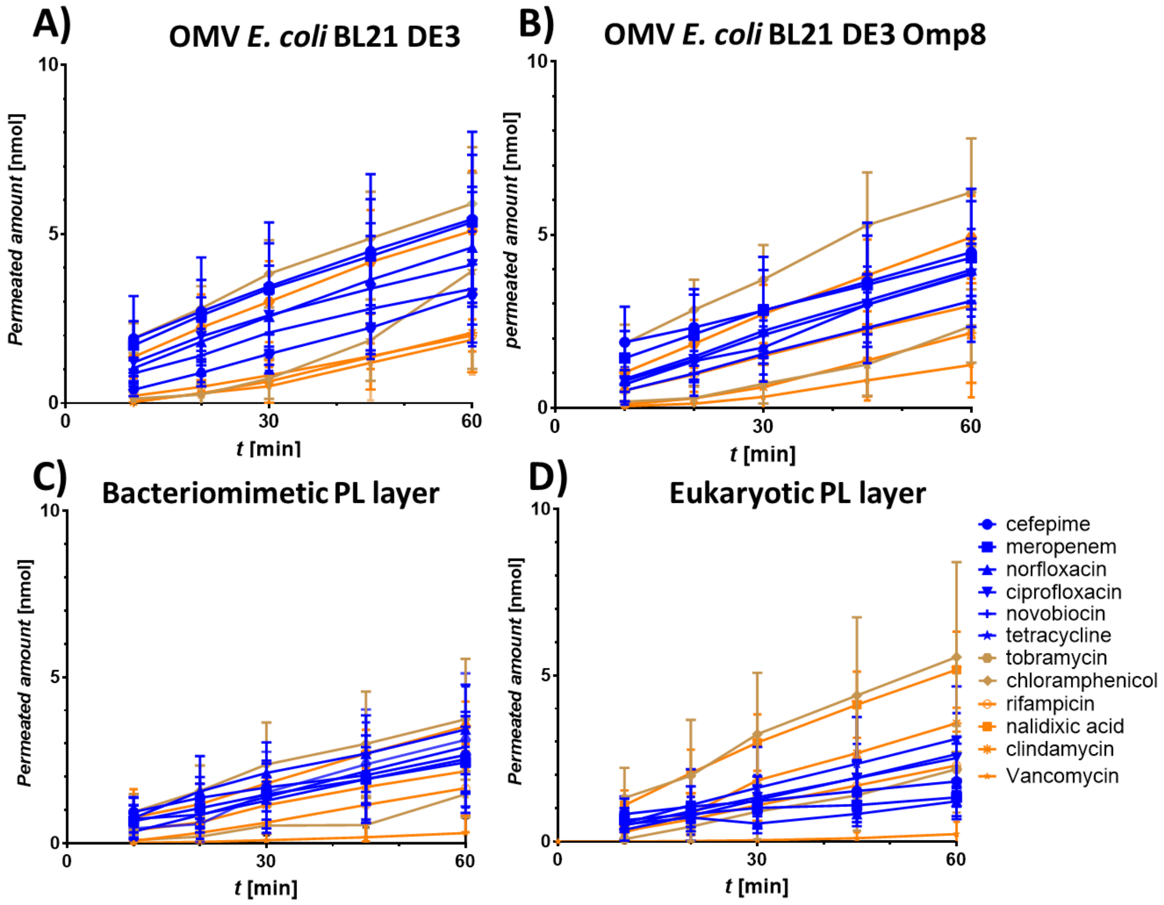

Figure S17. Permeation-time courses of eleven antibiotics obtained from membrane models derived from (A) outer membrane vesicles of *E. coli* BL21 DE3, (B) OMVs of *E. coli* BL21 DE3 Omp8, (C) bacteriomimetic liposomes and (D) eukaryotic liposomes.

Points represent mean + SD. N ≥ 9 from at least 3 independent experiments

Materials and Methods

Table S1. Absorbance wavelengths used for compound quantifications:

Compound	Wavelength [nm]
Ciprofloxacin	322
Nalidixic acid	330
Norfloxacin	324
Novobiocin	304
Tetracycline	372
Chloramphenicol	298
Cefepime	300
Meropenem	302

Table S2. LC–MS/MS parameters used for compound quantification:

Name	Flow rate [ml/min]	Column temp. [°C]	R_t [min]	Parent mass [m/z]	Fragment mass [m/z]	Collision Energy [V]	Tube Lens Offset	Spray voltage [V]	Vaporizer temp. [°C]	Capillary temp. [°C]	Sheath Gas Pressure	Ion Sweep Gas Pressure	Aux Gas Pressure	Column
Clindamycin	500	25	3.34	425.154	126.1<br>377.2<br>389.2<br>335.2	28<br>17<br>16<br>17	95	4500	300	300	35	0	10	Accucore^{TM} RP-MS, 150 mm x 2.1 mm, 2.6 µm (Thermo Fisher Scientific Co.)
Fusidic acid	300	25	6.34	539.400	479.4	18	124	5000	260	280	45	0	25	
Norfloxacin	300	25	2.28	320.1	302.2<br>231.1<br>276.2<br>282.2	20<br>40<br>16<br>29	100	4500	280	300	60	0	30	
Vancomycin	300	25	3.68	725.000	82.5<br>100.3<br>329.4<br>773.3<br>1306.0	65<br>17<br>30<br>39<br>14	95	3500	300	300	15	0	5	
Tobramycin	700	40	2.74	468.2	145.0<br>162.9<br>205.0<br>324.0	21<br>22<br>23<br>14	97	4000	400	240	50	0	30	Zorbax XDB-C18, 4.6 mm x 50 mm, 5µm (Agilent technolo-gies Inc.)
